# Microbubble-Templated Immunoactive Metal-Phenolic Capsules for Drug Delivery and Enhanced Cancer Immunotherapy

**DOI:** 10.34133/research.0752

**Published:** 2025-07-04

**Authors:** Xin Tan, Xiaojing Wu, Renwang Sheng, Yinghua Tao, Weikun Li, Yanling Liang, Bo Gui, Huiqin Lu, Diyi Feng, Nuoya Chen, Fangzhou Liu, Ling Liu, Liqin Ge

**Affiliations:** ^1^ State Key Laboratory of Digital Medical Engineering, School of Biological Science and Medical Engineering, Southeast University, Nanjing 210096, P.R. China.; ^2^ Jiangsu Provincial Key Laboratory of Critical Care Medicine, Department of Critical Care Medicine, Zhongda Hospital, School of Medicine, Southeast University, Nanjing 210009, P.R. China.; ^3^ School of Medicine, Southeast University, Nanjing 210009, P.R. China.; ^4^ Department of Head & Neck Surgery, Jiangsu Cancer Hospital & Jiangsu Institute of Cancer Research & The Affiliated Cancer Hospital of Nanjing Medical University, Nanjing 210029, P.R. China.; ^5^ Advanced Ocean Institute of Southeast University, Nantong 226000, P.R. China.

## Abstract

Advanced drug delivery systems integrating immunomodulatory functions can improve cancer therapy. Herein, we develop a novel metal-phenolic capsule for synergistic drug delivery and immunomodulation. Specifically, a stable metal-phenolic network (MPN), formed by Fe (III), tannic acid, and catechol-modified hyaluronic acid, was assembled onto doxorubicin (DOX)-loaded mannose-glycated bovine serum albumin microbubbles to construct microcapsules with both drug loading and immunomodulatory functions simultaneously. The capsules release DOX in a pH-responsive manner and induce reactive oxygen species accumulation in tumor cells, thereby enhancing immunogenic cell death (ICD). This ICD effect, combined with the direct stimulation of dendritic cells (DCs) by the capsules and the paracrine signaling from capsule-activated M1 macrophages, synergistically promotes DC maturation. In vivo bilateral tumor models demonstrate that DmTFH substantially inhibits primary and distant tumor development and markedly delays metastasis to the lungs. Flow cytometry analysis confirms robust local and systemic immune responses, characterized by enhanced DC maturation within lymph nodes and increased infiltration of activated CD8^+^ and CD4^+^ T cells in tumors and spleens. Overall, the immunoactive MPN microcapsules constructed in this study enhance the immunotherapeutic efficacy of DOX through the synergistic action of carrier-inherent immunomodulation and chemotherapy, providing a promising approach for combinatorial cancer therapy.

## Introduction

Doxorubicin (DOX), a Food and Drug Administration (FDA)-approved small-molecule anticancer drug used for the treatment of multiple cancer types, plays a vital role in cancer chemotherapy [1]. It induces the apoptosis of tumor cells through mechanisms involving DNA intercalation, topoisomerase II suppression, reactive oxygen species (ROS) production, and mitochondrial dysfunction [2]. Due to the high proliferation rate and heightened sensitivity to oxidative stress of breast cancer cells, DOX is widely used in clinical treatment for breast cancer. Recently, DOX garnered attention for its immunotherapeutic potential. It has been demonstrated to induce immunogenic cell death (ICD), a distinct form of cell death capable of triggering the host’s immune response [3]. A key feature of ICD is the secretion of certain signaling molecules by damaged or dying cells, which are known as damage-associated molecular patterns (DAMPs). Common DAMPs include high mobility group protein B1 (HMGB1), adenosine triphosphate (ATP), and calreticulin (CRT) exposed on the cell surface [4]. These DAMPs can act as “eat me” signals recognized by dendritic cells (DCs), promoting antigen uptake and presentation from dead tumor cells and thereby activating T cell-mediated adaptive immune responses. Research has shown that DOX is capable of inducing ICD across a range of tumor models, highlighting its potential for cancer immunotherapy [5]. Despite its therapeutic benefits, DOX faces significant challenges when used in tumor immunotherapy. The primary challenge is that ICD induced solely by DOX often fails to overcome the immunosuppressive tumor microenvironment, predominantly maintained by various immunosuppressive components such as anti-inflammatory M2 macrophages [6]. Therefore, developing combination strategies that synergistically enhance the immunotherapeutic effect of DOX has become an urgent requirement to maximize its therapeutic efficacy.

With advancements in biomaterial research, numerous biomaterials with immunomodulatory functions, including both natural and synthetic materials, have been implemented in cancer immunotherapy [7]. Compared to the direct use of immunomodulatory agents, delivery systems constructed from these biomaterials possess inherent immunomodulatory potential and can alter immune responses independently through their intrinsic active components, rather than relying on exogenous immunostimulants [8]. For instance, our recent study developed mannose-glycated metal-phenolic microcapsules capable of repolarizing anti-inflammatory M2 macrophages into pro-inflammatory M1 phenotypes and elevating the CD8^+^/CD4^+^ T cell ratio [9]. We hypothesize that integrating DOX with these inherently immunomodulatory microcapsules could reprogram the immunosuppressive tumor microenvironment and improve the insufficient systemic tumor antigen-specific cytotoxic T lymphocyte (CTL) responses associated with DOX monotherapy.

Drugs or therapeutic agents are typically encapsulated into microcapsules via 2 approaches, namely, pre-loading and post-loading [10]. The pre-loading method entails incorporating therapeutic compounds into sacrificial porous templates during the production of microcapsules to produce drug-loaded hollow structures [11]. In contrast, post-loading relies on modulating the permeability of pre-formed capsule walls for therapeutic cargo encapsulation. However, both methods face inherent limitations in effectively retaining small-molecule drugs, as these substances can freely diffuse through the capsule walls. To address this challenge, studies have developed a conjugation strategy where small molecules were linked to negatively charged carrier polymers, utilizing steric hindrance and charge repulsion to prevent the diffusion of encapsulated agents [12]. However, this approach requires the introduction of exogenous polymers and subsequent core dissolution processes to obtain hollow microcapsules containing the desired substance, significantly complicating the composition and preparation process of the delivery system.

In this study, we designed an immunoactive metal-phenolic capsule (referred to as DmTFH) loaded with DOX, which released DOX to induce ICD in cancer cells and interacted with immune cells, including DCs and macrophages, to reverse the immunosuppressive tumor microenvironment, thereby synergistically boosting cancer immunotherapy. First, DOX was adsorbed onto negatively charged mannose-glycated bovine serum albumin (mBSA) through electrostatic interactions. DOX-loaded mBSA microbubbles (MBs) were then generated via sonication and subsequently used as templates for capsule preparation. Next, a metal-phenolic network (MPN), formed by Fe^III^, tannic acid (TA), and catechol-modified hyaluronic acid (HADA), was assembled onto these MBs to enhance drug loading capacity and improve biocompatibility. After air spontaneously escaped from the MBs, DmTFH was successfully fabricated. The effects and underlying mechanisms of DmTFH in enhancing breast cancer cell death were further examined. Subsequently, the multiple pathways involved in DmTFH-mediated DC maturation were thoroughly explored. Finally, a bilateral tumor model was employed to evaluate the in vivo therapeutic effects of DmTFH, and flow cytometry analysis confirmed robust local and systemic immune responses induced by DmTFH. Collectively, this study presented a novel MPN-based capsule that synergistically combined chemotherapy and immunotherapy, leveraging the inherent immunomodulatory properties of the carrier to overcome the limitations of free DOX in immunotherapy.

## Results and Discussion

### Fabrication and characterization of DmTFH

**Fig. 1.**
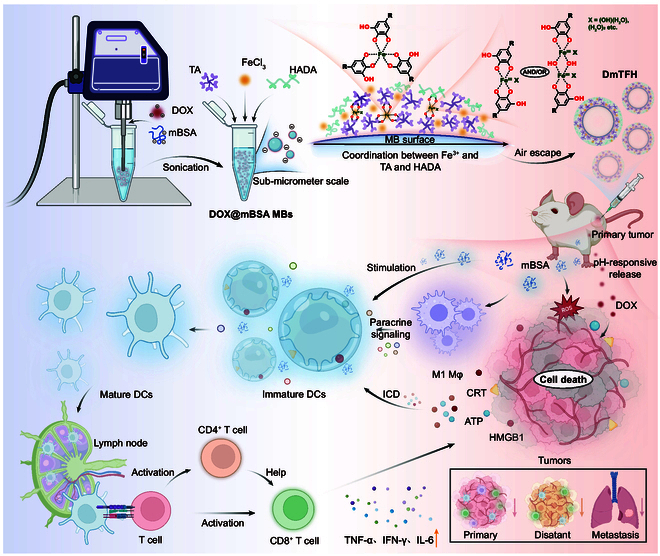
Schematic representation of the assembly steps of immunoactive metal-phenolic capsules templated by microbubbles, demonstrating the in situ loading of DOX and the MPN assembly mechanism on MBs’ surfaces, as well as the underlying mechanisms involved in tumor immunotherapy. Mφ, macrophage; TNF-α, tumor necrosis factor-α; IL-6, interleukin-6; IFN-γ, interferon-γ. The figure was created using BioRender (https://biorender.com/).

**Fig. 2.**
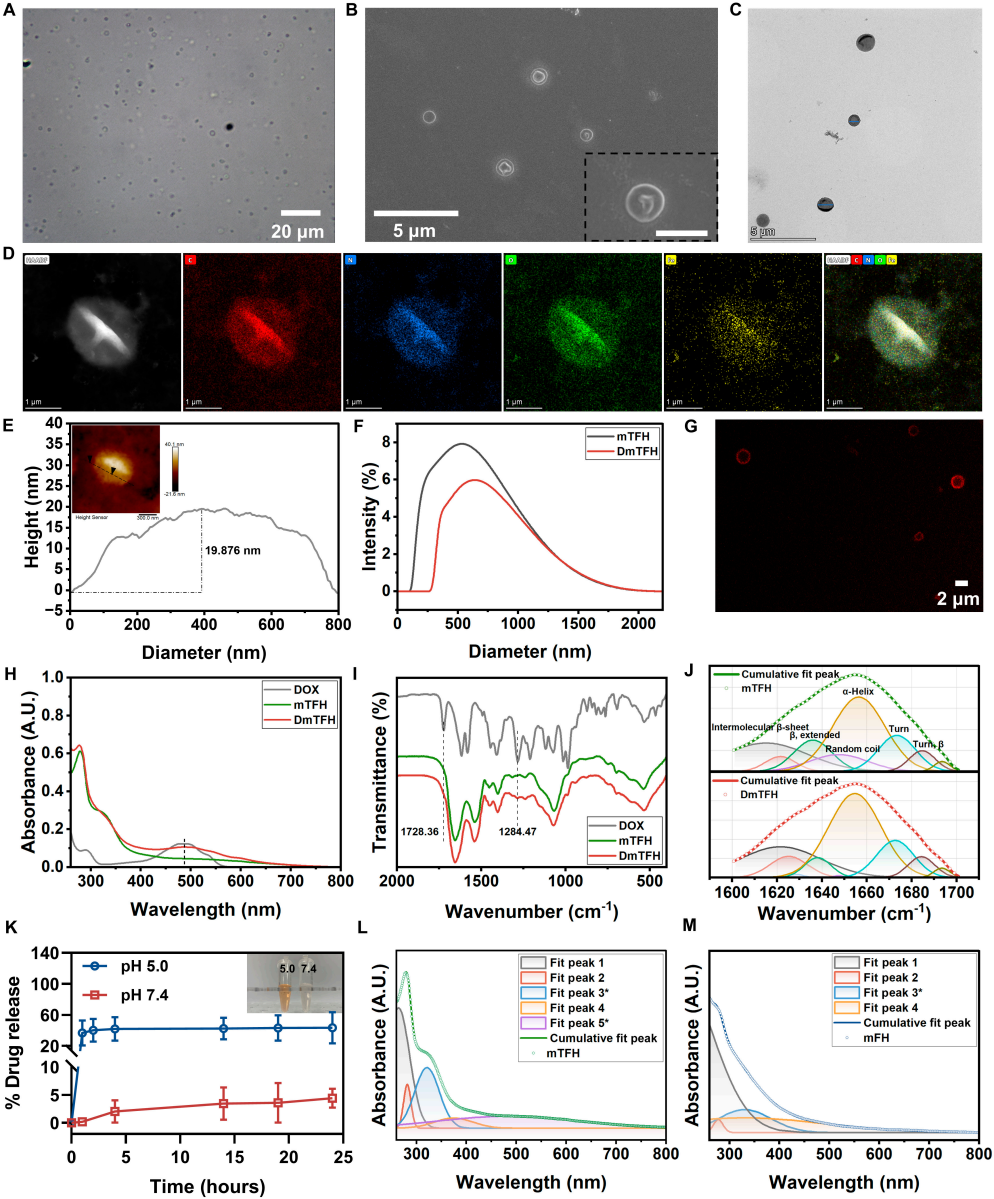
Fabrication and characterization of DmTFH. (A) OM, (B) SEM, (C) TEM image of DmTFH. The scale bar for the SEM image with a larger field of view was 1 μm. (D) HAADF and EDX elemental mapping of DmTFH. (E) AFM image of DmTFH and the corresponding height profile for an indicated area. (F) Size distribution of mTFH and DmTFH. (G) CLSM image of DmTFH. (H) UV–Vis and (I) FTIR spectra of DOX, mTFH, and DmTFH. (J) Secondary fitting curve of FTIR spectra of mTFH and DmTFH at the region of the amide I band (1,600 to 1,700 cm^−1^). (K) Drug release curves of DmTFH at different pH (*n* = 3). (L and M) UV–Vis spectra curve-fitting results of mTFH and mFH.

The preparation process of DmTFH was illustrated in Fig. 1. First, DOX was loaded onto mBSA through electrostatic interactions. This mixture was then subjected to repeated sonication to generate DOX@mBSA MBs. Next, the MPNs, formed by Fe (III), TA, and HADA (Fig. S1), were assembled on the surface of these MBs. The Fe (III) ions interacted with both TA and HADA to form a stable MPN network around the MBs. As the air inside the MBs escaped naturally, DOX and mBSA with therapeutic and immunomodulatory properties were retained within the capsule. This resulted in the formation of DOX-loaded metal-phenolic capsules (DmTFH) that simultaneously possessed both drug delivery and immunomodulatory capabilities. Optical microscopy (OM), scanning electron microscopy (SEM), and transmission electron microscopy (TEM) images of DmTFH were shown in Fig. 2A to C, indicating the successful fabrication of DmTFH. High-angle annular dark-field (HAADF) microscopy and energy-dispersive x-ray (EDX) spectroscopy revealed that the capsule primarily contained C, O, N, and Fe elements (Fig. 2D). The elemental quantitative analysis further showed the presence of 69.70 at % C, 14.65 at % N, 15.09 at % O, and 0.57 at % Fe in DmTFH (Fig. S2). The atomic force microscopy (AFM) image showed that the bilayer thickness of the dried capsule was 19.876 nm. Therefore, the corresponding shell thickness of the capsule was approximately 10 nm. The hydrodynamic diameter of DmTFH was about 650 nm with a polydispersity index of 0.488 based on the dynamic light scattering (DLS) analysis (Fig. 2F). Compared to mTFH (capsules without DOX loading), the DLS curve of DmTFH exhibited a slightly broader peak shifted to a larger diameter, indicating an increase in capsule size due to DOX loading. Zeta potential analysis indicated that after loading positively charged DOX, the surface potential of DmTFH slightly decreased (Fig. S3). Given the inherent fluorescence properties of DOX, the confocal laser scanning microscopy (CLSM) image showed that DmTFH exhibited red fluorescence, indicating the presence of DOX within the DmTFH (Fig. 2G). The characteristic peak at ~484 nm in the ultraviolet–visible (UV–Vis) spectrum of DmTFH confirmed that DOX has been successfully loaded into the capsules (Fig. 2H). We observed that the drug loading capacity of DmTFH increased with the initial DOX feed concentration, but the drug loading efficiency decreased as the feed concentration further increased (Fig. S4). Therefore, we chose an initial DOX feed concentration of 1 mg/ml as the optimal concentration for subsequent studies. At this feed concentration, each BSA molecule was conjugated with approximately 4 DOX molecules [1]. Fourier transform infrared spectrum (FTIR) showed characteristic peaks for DOX at 1,730 cm^−1^ (the vibration of aromatic aldehyde C=O) and 1,284 cm^−1^ (C–O–C vibrations) [13]. These peaks were also detected in DmTFH, indicating the presence of DOX within the DmTFH structure (Fig. 2I). The secondary fitting curves of FTIR reflected the conformational changes in mBSA after DOX loading (Fig. 2J). The result suggested that the electrostatic interactions between DOX and mBSA increased the stability of the mBSA molecule, as indicated by the decreased content of random coil and increased content of β-sheet and α-helix [14,15]. The charge effects also influenced the β-extended part, showing a red shift but with a decrease in content. The turn and β-turn parts showed no significant changes, and the detailed quantitative analysis was summarized in Table S1. In addition, MPNs were widely reported to exhibit pH-responsive characteristics [16,17]. When pH > 7, they formed tris-complexes; when pH was between 3 and 6, they formed bis-complexes; and when pH < 2, they disassembled into mono-complex forms. Thus, the pH-responsive behavior of DmTFH was further investigated (Fig. 2K). At a pH of 5.0, the release profile showed an initial burst, with a subsequent sustained release extending over 24 h, resulting in about 60% of the drug being released. However, the drug release was much lower at pH 7.4, with only about 5% drug release observed over the same period. The inset image showed the color change of the DmTFH solution at pH 5.0 and pH 7.4, indicating the pH-disassembly properties of the DmTFH. Moreover, we found that after 24 h in phosphate-buffered saline (PBS) solution (pH 7.4), DmTFH released less drug compared to DmFH, which was formed by only coating HADA and Fe^III^ on DOX-loaded mBSA MBs (Fig. S5A). The UV–Vis spectrum demonstrated that DmTFH had a significantly higher drug loading capacity compared to DmFH (Fig. S5B and C). These results implied that the MPN structure in DmTFH, formed by Fe (III), TA, and HADA, was more stable, leading to less drug release and increased drug loading capacity. Given these findings, we further investigated the assembly mechanism of Fe (III) with TA and HADA on the surface of MBs. The secondary fitting curve of UV–Vis spectra of mTFH showed 5 distinct peaks (Fig. 2L). Peak 1 can be attributed to the broad absorption of BSA in aqueous solution. Peak 2 was associated with the broad absorption of BSA and TA. Notably, peak 3 represented the conjugation between Fe^III^ and deprotonated phenolic hydroxyl groups. Peak 4 may be attributed to the broad absorption of Fe^III^ in aqueous solution, and peak 5 indicated the presence of tris-complexes, characterized by the ligand-to-metal charge transfer band (LMCT) [16,18,19]. The secondary fitting curve of mFH lacked a clear LMCT band showing only the conjugation between Fe^III^ and deprotonated phenolic hydroxyl groups of HADA (Fig. 2M). This was likely due to the steric hindrance effect of HADA, which prevented the formation of local tris-type complexes [18]. These results confirmed that the stable MPNs formed by Fe^III^, TA, and HADA on MBs were primarily composed of tris-complexes formed between TA and Fe^III^, along with conjugations between Fe^III^ and deprotonated phenolic hydroxyl groups of both TA and HADA (Fig. 1).

### Antitumor effect of DmTFH in vitro

**Fig. 3.**
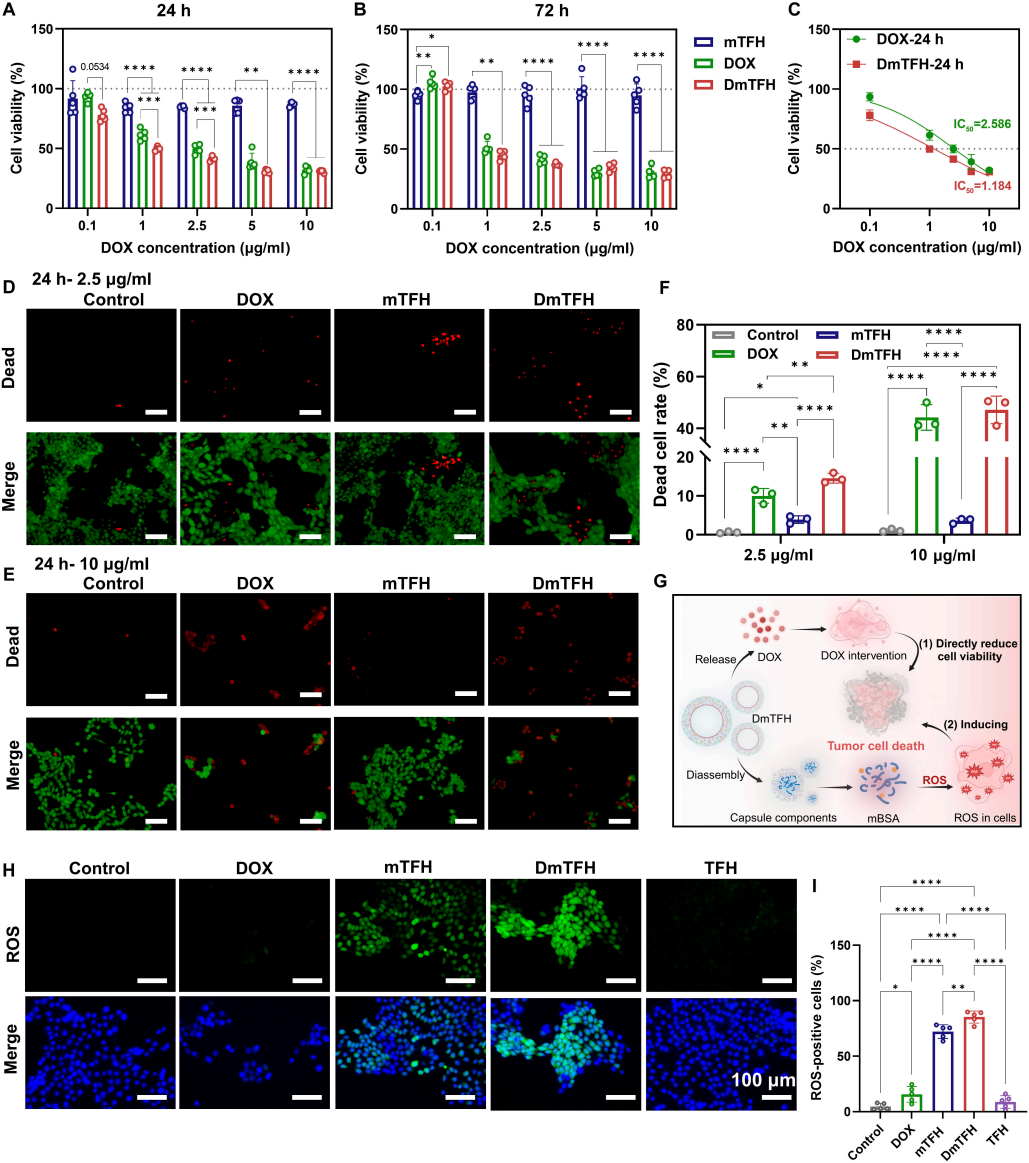
Antitumor effect of DmTFH in vitro. (A) Cell viability of 4T1 cells following treatment with varying doses of free DOX, mTFH, and DmTFH for 24 h and (B) 72 h (*n* = 5). (C) IC_50_ of DOX and DmTFH against 4T1 cells after a 24-h incubation period (*n* = 5). (D and E) Images of live/dead staining of 4T1 cells following a 24-h incubation with free DOX, mTFH, and DmTFH at corresponding concentrations for 24 h. Control represents untreated cells. Green and red fluorescence indicate live cells and dead cells, respectively. Scale bar, 100 μm. (F) Quantitative analysis of dead cell rates in 4T1 cells after incubation with free DOX, mTFH, and DmTFH at corresponding concentrations for 24 h (*n* = 3). (G) Illustration of the antitumor mechanism of low-concentration DmTFH. (H) ROS staining images of 4T1 cells after incubation with free DOX, mTFH, DmTFH, and TFH at a concentration of 2.5 μg/ml for 24 h. Control represents untreated cells. Green fluorescence represents ROS-positive cells, while blue fluorescence indicates cell nuclei. Scale bar, 100 μm. (I) Corresponding quantitative analysis of ROS-positive cells in 4T1 cells (*n* = 5). (G) was created using BioRender (https://biorender.com/).

After the successful loading of DOX, we further assessed the in vitro therapeutic effect of DmTFH. First, the standard cell counting kit-8 (CCK-8) assay result showed that drug treatment markedly decreased the survival rate of 4T1 cells. Notably, DmTFH exhibited higher cytotoxicity against 4T1 cells compared to the DOX group alone at 24 h with DOX concentrations ranging from 0.1 to 2.5 μg/ml (Fig. 3A and C). However, with the increase in concentration or incubation time, there was no marked variation in cell viability observed among the 2 groups (Fig. 3A and B). To further assess cell death under different drug concentrations, live/dead cell staining was performed. Consistent with the CCK-8 results, the staining results demonstrated that both free DOX and DmTFH significantly induced tumor cell death at a high concentration (10 μg/ml), as indicated by the high proportion of dead cells (Fig. 3E and F). At a lower concentration (2.5 μg/ml), the DmTFH group exhibited a higher number of dead cells (indicated by red fluorescence) compared to the DOX-only group after 24 h (Fig. 3D and F). Notably, at the same concentration, no substantial cell death was observed in the free DOX group at either 24 or 72 h, although the CCK-8 assay revealed a marked decrease in cell viability (Fig. 3D and Fig. S6). Since the cell viability measured by the CCK-8 assay reflected a combination of inhibited cell proliferation and cell death, we hypothesized that low-dose DOX mainly exerted its cytotoxic effect by suppressing cell proliferation. This was supported by the observation of a significant reduction in the number of living cells in the DOX-treated groups at 72 h (Fig. S6). These findings suggested that, at this concentration, free DOX primarily exerted its cytotoxic effects by inhibiting cell proliferation, which was consistent with previous reports [20]. The combination treatment with capsules can enhance the antitumor efficacy of low-concentration DOX by simultaneously inhibiting cell proliferation and promoting cell death. Studies have shown that breast cancer cells are highly sensitive to ROS, making these cells prone to lethal oxidative stress [21]. Previous research has demonstrated that DOX can enter mitochondria and capture an electron from the mitochondrial electron transport chain (mETC), resulting in the formation of a semiquinone intermediate. This intermediate subsequently transfers the electron to O₂ through redox cycling, ultimately leading to the production of ROS [2,22,23]. Our previous study and other studies have also reported that glycated proteins can elevate ROS levels by activating intracellular signaling pathways or impairing mitochondrial function [9,24,25]. Therefore, we speculated that the enhanced anticancer efficacy of low-concentration DmTFH might be attributed to elevated intracellular ROS levels. To test this, ROS accumulation in 4T1 cells was measured using the DCFH-DA (2′,7′-dichlorofluorescin diacetate) probe following different treatments. As illustrated in Fig. 3H and I, the DOX group exhibited only a slight increase in ROS production compared to the control group. This phenomenon has also been reported in other studies, primarily since the preferential accumulation of free DOX in the nucleus instead of in the mitochondria prevents DOX molecules from efficiently capturing electrons from the mETC, and the hypoxic tumor microenvironment limits the availability of sufficient O_2_ for redox cycling-mediated ROS production [23]. In contrast, substantial ROS-positive cells were detected in groups treated with glycated protein-containing capsules (mTFH and DmTFH). To verify the essential role of the mannose-glycated BSA in ROS generation, the fluorescence from cells treated with the non-glycated capsule (TFH) was also detected. The results showed that only faint green fluorescence was observed, indicating TFH’s limited ability to induce ROS. This limitation could be attributed to the small amount of Fe^3+^ present on the capsule’s shell, which was insufficient for effective chemodynamic therapy to generate ROS (Fig. S2). These results indicated that the superior antitumor efficacy of low-concentration DmTFH was ascribed to the synergistic action of glycated capsules and DOX. On the one hand, DOX released from the capsule can directly reduce tumor cell viability. On the other hand, mannose-glycated BSA in the capsule induced ROS generation in 4T1 cells, thereby enhancing oxidative stress in tumor cells (Fig. 3G).

### Effects of DmTFH on in vitro ICD induction and immunostimulation

**Fig. 4.**
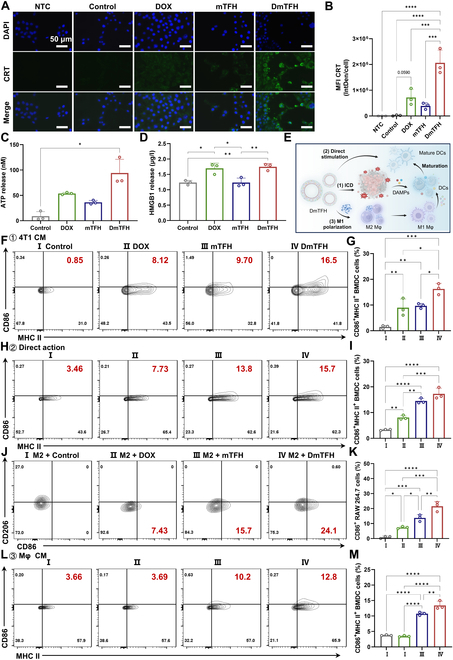
Effects of DmTFH on in vitro ICD induction and immunostimulation. (A) Immunofluorescence staining of 4T1 cells after 24-h incubation with different treatments. Cells were stained for CRT (green) and nuclei (DAPI, blue). Scale bar, 50 μm. (B) Quantification of CRT expression on the surface of 4T1 cells (*n* = 3). (C) ATP release from 4T1 cells after 24-h incubation with different treatments (*n* = 3). (D) HMGB1 release from 4T1 cells after 24-h incubation with different treatments, as evaluated by ELISA (*n* = 3). (E) Illustration of DC maturation promoted by DmTFH through multiple pathways, including (1) DOX-induced ICD with ROS-enhanced DAMP release and exposure; (2) carrier-mediated direct DC activation; (3) mBSA-driven M2 to M1 repolarization of macrophage for indirect DC stimulation. (F) Flow cytometry analysis of BMDCs after coculture with supernatants from 4T1 cells treated with different conditions. (G) Quantification of CD86^+^MHC II^+^ BMDCs after coculture with supernatants from 4T1 cells treated with different conditions (*n* = 3). (H) Flow cytometry analysis of BMDCs after direct culture with different treatments. (I) Quantification of CD86^+^MHC II^+^ BMDCs after direct culture with different treatments (*n* = 3). (J) Flow cytometry analysis of M1 polarization in M2-type macrophages following incubation with different treatments. (K) Quantification of the number of M1 (CD86^+^) macrophages after different treatments in vitro (*n* = 3). (L) Flow cytometry analysis of BMDCs after coculture with CM from RAW 264.7 cells treated with different conditions. (M) Quantification of CD86^+^MHC II^+^ BMDCs after coculture with CM from RAW 264.7 cells treated with different conditions (*n* = 3). (E) was created using BioRender (https://biorender.com/).

DOX has been widely reported to promote the exposure or release of DAMPs from tumor cells, such as CRT, ATP, and HMGB1. These danger signals can subsequently trigger the immune system to elicit tumor-specific cytotoxicity. In addition, ROS plays a pivotal role in anthracycline-induced ICD, such as facilitating the exposure of CRT on the cell surface [26]. Therefore, we evaluated whether DmTFH treatment may elicit ICD in tumor cells and enhance the release of DAMPs. First, we used immunofluorescence staining to evaluate the transfer of CRT protein from the endoplasmic reticulum to the cell surface after low-dose drug treatment. Immunofluorescence images revealed that low concentrations of DOX-treated cells showed only a small amount of CRT efflux, evident from the faint green fluorescence observed on the 4T1 cell surface (Fig. 4A). In contrast, a notable increase in CRT expression was detected on 4T1 cells following 24 h of exposure to DmTFH. Further quantitative assessment revealed that free DOX was also able to induce CRT expression compared to the control group, although the difference did not reach statistical significance (*P* = 0.0590). Notably, 4T1 cells treated with DmTFH displayed a mean fluorescence intensity (MFI) of CRT expression twice as high as that in the DOX group, indicating that DmTFH more effectively promoted CRT exposure, benefiting from the combined effects of DOX and ROS (Fig. 4B). Additionally, we evaluated the release of ATP and HMGB1. The results showed that ATP release from 4T1 cells was significantly increased following DmTFH treatment compared to the control group (Fig. 4C and Fig. S7). Moreover, both DOX- and DmTFH-treated cells exhibited a marked increase in HMGB1 release relative to the control and mTFH-treated groups (Fig. 4D and Fig. S7). However, no significant difference was observed between the DmTFH and free DOX groups, suggesting that HMGB1 release induced by DmTFH was primarily attributable to the effect of the loaded DOX. Collectively, these findings suggested that while free DOX alone can induce certain levels of ICD, its combination with the capsule significantly amplified CRT exposure and ATP release, thereby potentially enhancing the overall immune response. To further evaluate the immune response mediated by ICD, bone marrow-derived dendritic cells (BMDCs) were isolated (Fig. S8) and subsequently cocultured with the supernatants of 4T1 cells that had been treated with various groups. The supernatants contained DAMPs and debris from the 4T1 cells, which can promote DC maturation. Therefore, we conducted the flow cytometric analysis to assess DC maturation in each treatment group by labeling major histocompatibility complex class II (MHC II) and CD86. DmTFH was more effective in inducing DC maturation compared to other groups, with a significant increase to 16.5% of mature DCs (Fig. 4F and G). This indicated that the DmTFH-mediated ICD effect promoted the maturation of DCs. Notably, in addition to contacting tumor cells, drug carriers in the tumor microenvironment may directly act on immune cells. Therefore, low-concentration DmTFH (DOX concentration: 1 μg/ml) was incubated directly with immature BMDCs to study their direct immunostimulatory effects. We unexpectedly found that the proportion of mature BMDCs was approximately 13.8% in the mTFH group, representing a 1.8-fold increase compared to the group treated with free DOX (Fig. 4H and I). This finding may be attributed to mBSA in the capsule, as glycated BSA has been reported to induce DC maturation, as shown by heightened levels of CD1a, CD40, CD80, CD83, CD86, and MHC class II molecules [27]. Among the tested formulations, the highest proportion of CD86^+^MHC II^+^ BMDCs was detected in the DmTFH group, indicating its strong direct immunostimulatory effect on BMDCs due to the synergistic action of DOX and mBSA. Furthermore, numerous studies have shown that M1 macrophages facilitate DC maturation and boost antitumor immunity by secreting related paracrine signaling [28,29]. Our previous research found that glycated proteins can influence macrophage polarization toward the M1 phenotype via the activation of the nuclear factor κB (NF-κB) pathway [9]. Thus, we further investigated whether DmTFH could indirectly promote the maturation of DCs by modulating M1 polarization of macrophages. After treating RAW 264.7 cells (mouse macrophages) with interleukin-4 (IL-4) and IL-13 to induce an M2 phenotype (typical morphology shown in Fig. S9), we evaluated the ability of different treatment groups to induce M2-to-M1 repolarization in macrophages using flow cytometry. In contrast to the control and DOX groups, a higher number of CD86^+^ cells were detected in the mTFH group, suggesting that the capsule alone can induce macrophage polarization toward the M1 phenotype. Notably, the positive expression rate of CD86 in cells treated with DmTFH was approximately 24.1%, confirming that DmTFH significantly promoted a transition from the M2 to the M1 activation state in macrophages (Fig. 4J and K). Subsequently, CM from macrophages treated with different groups were collected to study their impact on BMDC maturation. It was observed that comparable levels of mature DCs were present in both the control and DOX groups, indicating that free DOX’s weak activation of M1 macrophages failed to significantly promote BMDC maturation (Fig. 4L). Conversely, CM from mTFH and DmTFH treatments effectively induced DC maturation, with proportions of 10.2% and 12.8%, respectively (Fig. 4L and M). This suggested that the paracrine signals from DmTFH-activated M1 macrophages can further promote DC maturation. Overall, these results revealed that DmTFH can mediate BMDC maturation through multiple pathways (Fig. 4E). First, DmTFH relied on DOX to cause ICD in cancer cells and synergized with ROS to enhance ICD effects. DAMPs triggered by ICD can increase the levels of mature DCs. Second, DmTFH directly promoted DC maturation due to the immunomodulatory effects of the carrier itself. Additionally, DmTFH can indirectly stimulate DC activation by regulating immunosuppressive immune cells (M2 macrophages). The combined action of these mechanisms made DmTFH exhibit promising immunostimulating effects, thus having potential in vivo antitumor applications.

### In vivo antitumor effects of DmTFH

**Fig. 5.**
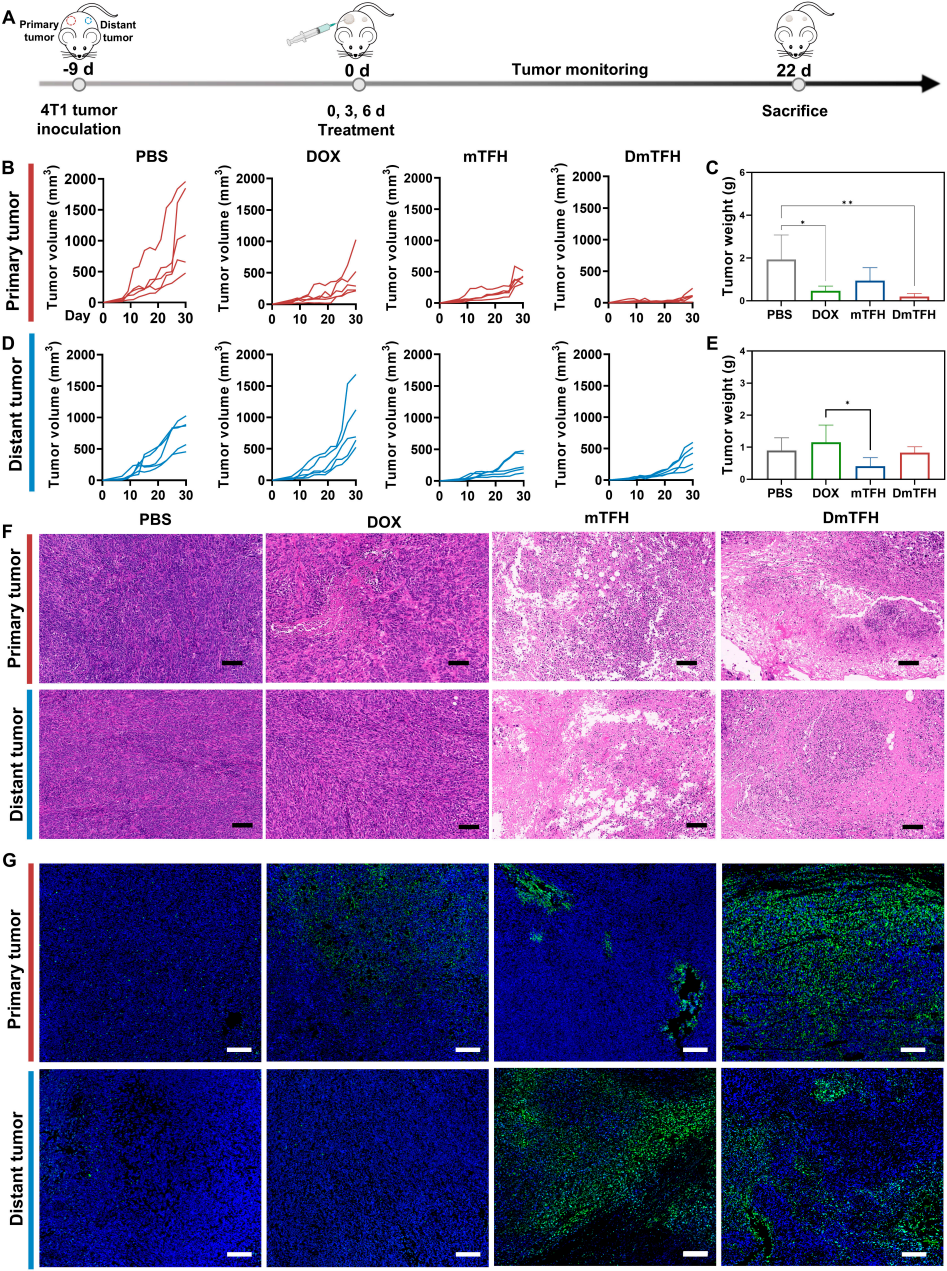
In vivo antitumor effects of DmTFH. (A) Schematic representation of the establishment of a bilateral tumor model and the subsequent treatment process. (B) Growth curves of primary and (D) distant tumors over 22 d following intratumoral injection of PBS (control), DOX, mTFH, or DmTFH (*n* = 5). (C) Final weights of the excised primary and (E) distant tumors (*n* = 5). (F) H&E staining of primary and distant tumors from different treatment groups. Scale bars, 100 μm. (G) TUNEL staining of primary and distant tumors from different treatment groups. Apoptotic cells appear as green fluorescence. Scale bars, 100 μm.

Subsequently, the in vivo antitumor effects of DmTFH were explored. For this purpose, we established a bilateral tumor model by implanting 4T1 tumor cells on both sides of the dorsal region (Fig. 5A). Nine days later, all tumor-bearing mice were randomly divided into 4 groups, and different formulations were intratumorally injected into the primary tumor site. The body weight of the mice and the sizes of the bilateral tumors for each treatment group were monitored. The growth curves of bilateral tumors in each mouse were depicted in Fig. 5B and D, while the final weights and images of excised tumors post-treatment were shown in Fig. 5C and E and Fig. S10. Compared to other groups, DmTFH treatment resulted in a significant reduction in the development of the primary tumor, with average tumor sizes of 114.61 mm^3^ (Fig. 5B). The superior therapeutic effect of the DmTFH group in treating the primary tumor reflected the enhanced local chemotherapeutic effect of DOX. DmTFH enabled the release of DOX at the primary tumor site, inducing significant cytotoxicity and thereby more effectively suppressing the growth of the primary tumor. However, although DOX treatment inhibited the rapid development of primary tumors in mice due to its direct cytotoxic effects on tumor cells, the suppression of distant tumors was less effective, with an average tumor volume as high as 930.82 mm^3^, even exceeding that of the PBS group. The growth of distant tumors reflected the level of systemic immune activation. The volume of distant tumors in the DOX group was larger than that in the PBS group, suggesting that DOX treatment might induce systemic immunosuppression, which was confirmed by subsequent systemic immune analyses. The progression of both proximal and distal tumors in mice treated with mTFH was also suppressed, with average tumor volumes of 397.00 and 289.64 mm^3^, respectively. Subsequently, the bilateral tumors from each experimental group were recorded by weighing and photographing. The DmTFH group exhibited the smallest primary tumors, with an average weight of 0.196 g, revealing its potent antitumor capability (Fig. 5C and Fig. S10). Unexpectedly, the distant tumors from the mTFH group were the lightest, with an average weight of 0.41 g, indicating the significant impact of mTFH on strengthening immune-mediated antitumor effects (Fig. 5E and Fig. S10). The slightly larger volume of distant tumors in the DmTFH group compared to the mTFH group may be attributed to the mild immunosuppressive side effects caused by released DOX, which could weaken systemic immune activation. This observation was consistent with the finding that the distant tumor volume in the DOX group was greater than that in the PBS group, indicating that DOX may induce systemic immunosuppression in vivo. Furthermore, the histological and apoptotic changes in tumor tissues following different treatments were visualized by H&E (hematoxylin and eosin) and TUNEL (terminal deoxynucleotidyl transferase–mediated deoxyuridine triphosphate nick end labeling) staining. As displayed in Fig. 5F and G, a significantly reduced tumor cell density was observed in bilateral tumors of both mTFH and DmTFH groups. Although some disruption of primary tumors was noted in the DOX group, the distant tumor tissue showed significant nuclear infiltration of tumor cells, with almost no apoptotic cells detected. The TUNEL staining combined with the quantification analysis further confirmed that DmTFH induced the highest level of apoptosis in primary tumor tissues, indicating its strong antitumor effect (Fig. 5G and Fig. S11). A substantial number of green fluorescence spots also appeared in the sections of distant tumors in the mTFH group, consistent with the results of the tumor growth curve. The stronger treatment effect for distant tumors than primary tumors in the mTFH group suggested that the distant tumor was more sensitive to the systemic immune response activated by mTFH. This could be attributed to a relatively weaker immunosuppressive microenvironment in the distant tumor, making it more susceptible to infiltration by immune effector cells. In the tumor model, primary and distant tumors were established by injecting different numbers of 4T1 cells, resulting in a larger tumor burden in the primary site and a smaller one in the distant site. Extensive preclinical and clinical evidence has demonstrated that, at both local and systemic levels, larger tumors exhibit stronger immunosuppressive properties than smaller ones, which can impair the immune responses elicited by immunotherapy [30]. In summary, these results highlighted the effectiveness of DmTFH in delaying tumor growth, underscoring its potential as a promising therapeutic strategy for cancer. Additionally, leveraging the immunomodulating properties of the capsule itself, DmTFH may improve the poor effects of free DOX on activating the immune system.

### In vivo ICD effect and immune activation effect of DmTFH

**Fig. 6.**
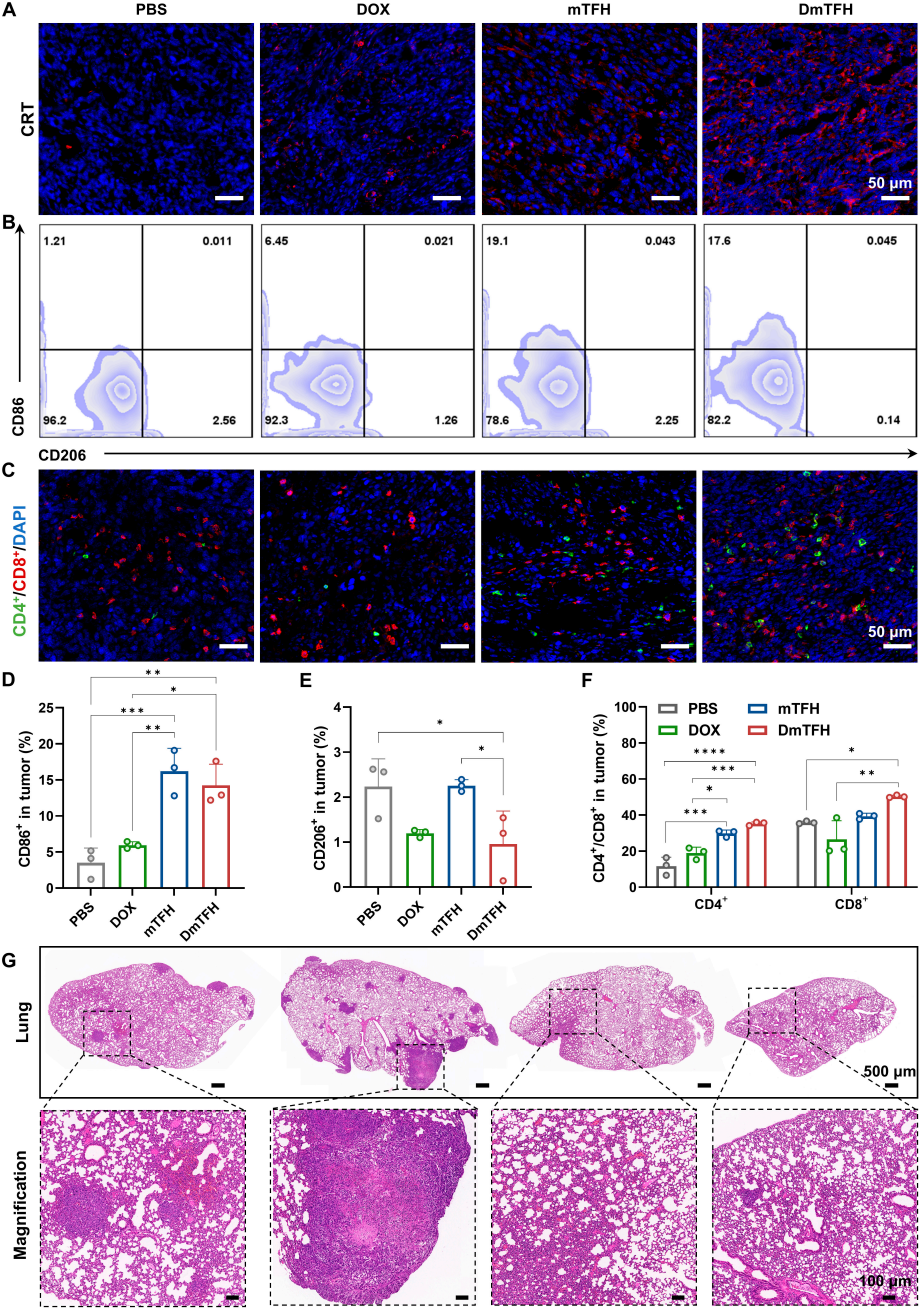
In vivo ICD effect and immune activation effect of DmTFH in tumors. (A) CRT (red) and DAPI (blue) immunofluorescence staining in tumor tissues from PBS-, DOX-, mTFH-, and DmTFH-treated mice. Scale bar, 50 μm. (B) CD86^+^ and CD206^+^ macrophage analysis via flow cytometry in tumor tissues from different treatment groups. (C) CD4^+^ (green) and CD8^+^ (red) T cell immunofluorescence staining in tumor tissues from different groups. Scale bar, 50 μm. (D) Flow cytometry quantification of the percentage of CD86^+^ and (E) CD206^+^ cells in tumor tissues. (F) Flow cytometry quantification of CD4^+^ and CD8^+^ T cells in tumor tissues. (G) H&E-stained lung sections from mice in the indicated groups. Scale bars, 500 μm and 100 μm.

Given the promising inhibitory effects of DmTFH on bilateral tumors observed in the tumor treatment experiments, we proceeded to assess its impact on the immune system. Initially, we performed immunofluorescence staining for CRT protein in tumor tissues to assess the efficacy of ICD induced by different treatments. The results revealed a significant red fluorescence signal in the primary tumors of the DmTFH group, confirming DmTFH’s ability to trigger ICD in tumor cells in vivo (Fig. 6A). However, only partial red fluorescence was detected in the tumors of the DOX group, suggesting that the ICD effects mediated by DOX were limited in vivo. This observation aligned with previous findings reported in the literature [31,32]. Surprisingly, mTFH also exhibited some ability to induce ICD, as evidenced by the moderate red fluorescence observed in the tumor tissues. These findings largely aligned with the previously mentioned tumor suppression results. Next, we evaluated the infiltration of key immune cells within the tumor. The tumor microenvironment often contains M2-phenotype macrophages, which are immunosuppressive and can promote tumor growth while inhibiting the immune system’s ability to target and destroy tumor tissue. Extensive research has reported that triggering ICD in tumor cells and releasing DAMPs can prompt macrophage polarization within the tumor from an M2 to an M1 state [33,34]. Furthermore, our study found that glycation capsules can enhance interactions with macrophages, promoting the accumulation of intracellular inflammatory mediators to modulate the repolarization of macrophages from M2 to M1 [9]. These M1-type macrophages helped improve the tumor’s immunosuppressive microenvironment and activated the immune system to specifically target and destroy tumor cells. Therefore, we further investigated the M1 polarization status of macrophages within tumor tissues after different treatments. The percentage of CD86^+^ macrophages in the tumors of mice treated with mTFH and DmTFH increased significantly to 19.1% and 17.8%, respectively, which was more than a 4-fold increase over the control group (Fig. 6B and D). Moreover, the percentage of CD206^+^ cells in the DmTFH group markedly decreased (Fig. 6E). These findings indicated that DmTFH can effectively stimulate the polarization of tumor-associated macrophages from an M2 to an M1 phenotype, mainly benefiting from the role of mTFH. Furthermore, we assessed T cell infiltration in tumor tissues. CD4^+^ helper T cells and CD8^+^ cytotoxic T cells represent 2 crucial subtypes of T lymphocytes. Cytotoxic T cells are capable of identifying and eliminating tumor cells directly, while helper T cells assist in this process [35]. Immunofluorescence analysis of tumor tissue sections revealed that, relative to the control group, mice treated with DmTFH showed a marked enhancement in the intensity of green (CD4^+^) and red (CD8^+^) fluorescence signals. This indicated that DmTFH-induced immune activation can lead to an enhancement of both CD4^+^ and CD8^+^ T cell infiltration in tumors, ultimately contributing to tumor cell killing (Fig. 6C). In contrast, the DOX group showed only a slight increase in green fluorescence, suggesting that DOX had limited capacity to induce immune stimulation in vivo. The mTFH group also displayed a substantial increase in both green and red fluorescence signals, confirming that the capsule carrier itself possessed intrinsic immunostimulatory properties for T cell activation. Flow cytometry quantification indicated that, compared to treatments with PBS, DOX, and mTFH, DmTFH administration markedly enhanced the presence of both CD4^+^ and CD8^+^ T cells within the tumor microenvironment. Specifically, the proportions reached 35.30% for CD4^+^ T cells and 50.3% for CD8^+^ T cells (Fig. 6F). These findings demonstrated that DmTFH was highly effective in enhancing the immune response within tumors. We further observed differences in tumor metastasis in lung tissue sections from mice treated with different therapies. The DmTFH-treated group displayed the least amount of tumor infiltration and metastasis among all treatment groups (Fig. 6G). The high-magnification images provided a clearer view of the tumor cells within the lung tissues. These results suggested that the effective immune response triggered by DmTFH prominently delayed the progression of lung metastasis.

**Fig. 7.**
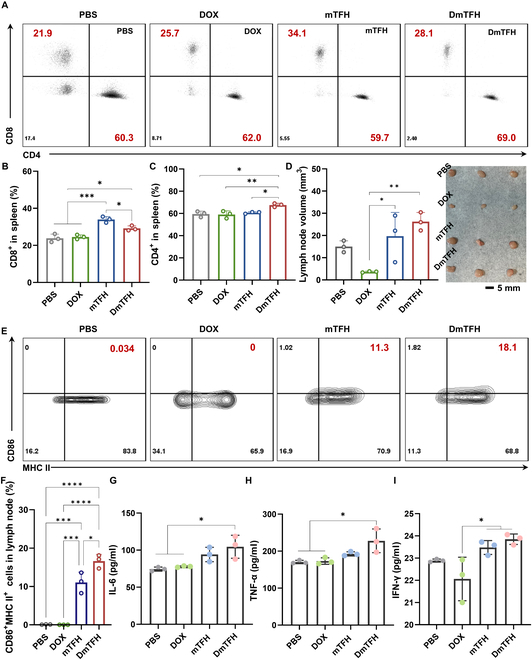
In vivo systemic immune activation effect of DmTFH. (A) Flow cytometry analysis of CD8^+^ and CD4^+^ T cell populations in the spleen following different treatments. (B) Quantification of CD8^+^ and (C) CD4^+^ T cells in the spleen (*n* = 3). (D) Lymph node volume measurements after various treatments (*n* = 3). The inset shows representative images of lymph nodes. (E) Evaluation of DC maturation in lymph nodes using flow cytometry, based on the expression levels of CD86 and MHC II. (F) Quantification of CD86^+^MHC II^+^ DCs in lymph nodes (*n* = 3). (G) ELISA measurement of IL-6, (H) TNF-α, and (I) IFN-γ levels in serum (*n* = 3).

Motivated by the robust immune activation at the primary tumor site, we explored how DmTFH triggers systemic immune reactions. As key immune organs, the spleen and lymph nodes were specifically examined for signs of immune activation. Flow cytometry was employed to assess the T cell phenotype in the spleen and the maturation status of DCs in the lymph nodes. The strategy for gating to identify T cell populations and mature DCs was demonstrated in Figs. S12 and S13. As presented in Fig. 7A, a noticeable rise in CD8^+^ T cell frequency was observed in the spleens of mTFH group-treated mice, accounting for 34.1%. This observation aligned with our previous report showing that the carrier itself has the capacity to activate T cells through its intrinsic active component, mBSA [9]. Due to the absence of potential immunosuppressive side effects associated with DOX, the systemic immune response triggered by mTFH appeared to be stronger than DmTFH, showing better performance in T cell activation. This could be the crucial reason for significant distant tumor suppression and delayed lung metastasis observed in the mTFH group in Figs. 5E and 6G. Consequently, owing to the intrinsic immunomodulatory effect of the carrier, the DmTFH group showed markedly higher proportions of CD4^+^ and CD8^+^ T cells (69.0% and 28.1% in the spleen) compared to the free DOX group (Fig. 7B and C). These results demonstrated that intratumoral injection of DmTFH elicited a systemic immune response, which was pivotal in suppressing distant tumor growth and enhancing overall tumor treatment efficacy. Further observations revealed differences in the sizes of the inguinal lymph nodes following different treatments. Surprisingly, we observed that DOX-treated mice had smaller lymph node volumes compared to the PBS group, a finding consistent with a previous study reporting severe tissue damage and splenic contraction in DOX-treated mice [31]. This phenomenon was potentially linked to DOX-induced immunosuppression due to its undifferentiated toxicity toward immune cells. In comparison to the DOX group, both mTFH and DmTFH led to a significant enlargement of the inguinal lymph nodes, demonstrating a strong immune response (Fig. 7D). Analysis via flow cytometry of mature DCs in the lymph nodes revealed that mTFH and DmTFH treatments resulted in a notable increase in the percentage of CD86^+^MHC II^+^ DCs (11.3% and 18.1%) compared to the DOX-treated group (0%) (Fig. 7E and F). Additionally, the cytokine levels in mouse serum after various treatments were assessed using enzyme-linked immunosorbent assay (ELISA) tests to further characterize the systemic immunostimulating effects of DmTFH. The findings showed a marked rise in IL-6, tumor necrosis factor-α (TNF-α), and interferon-γ (IFN-γ) levels in the serum of DmTFH-treated mice relative to the control group (Fig. 7G to I). Especially, IFN-γ levels in the serum of the DOX group were lower than the PBS group, confirming that DOX treatment may cause systemic immunosuppression in vivo. Collectively, DmTFH demonstrated superior tumor suppression at the primary tumor site due to the chemotherapeutic effect of DOX, whereas mTFH exhibited better control of distant tumors and improved performance in certain immune indicators owing to its intrinsic immunostimulatory capability. These findings reflected the synergistic mechanisms between chemotherapy and immunotherapy and highlighted the local and systemic immune-activating effects of DmTFH compared to free DOX, indicating that DmTFH treatment can effectively reprogram the suppressive tumor microenvironment, thus boosting antitumor immunity.

### In vivo biocompatibility of DmTFH

**Fig. 8.**
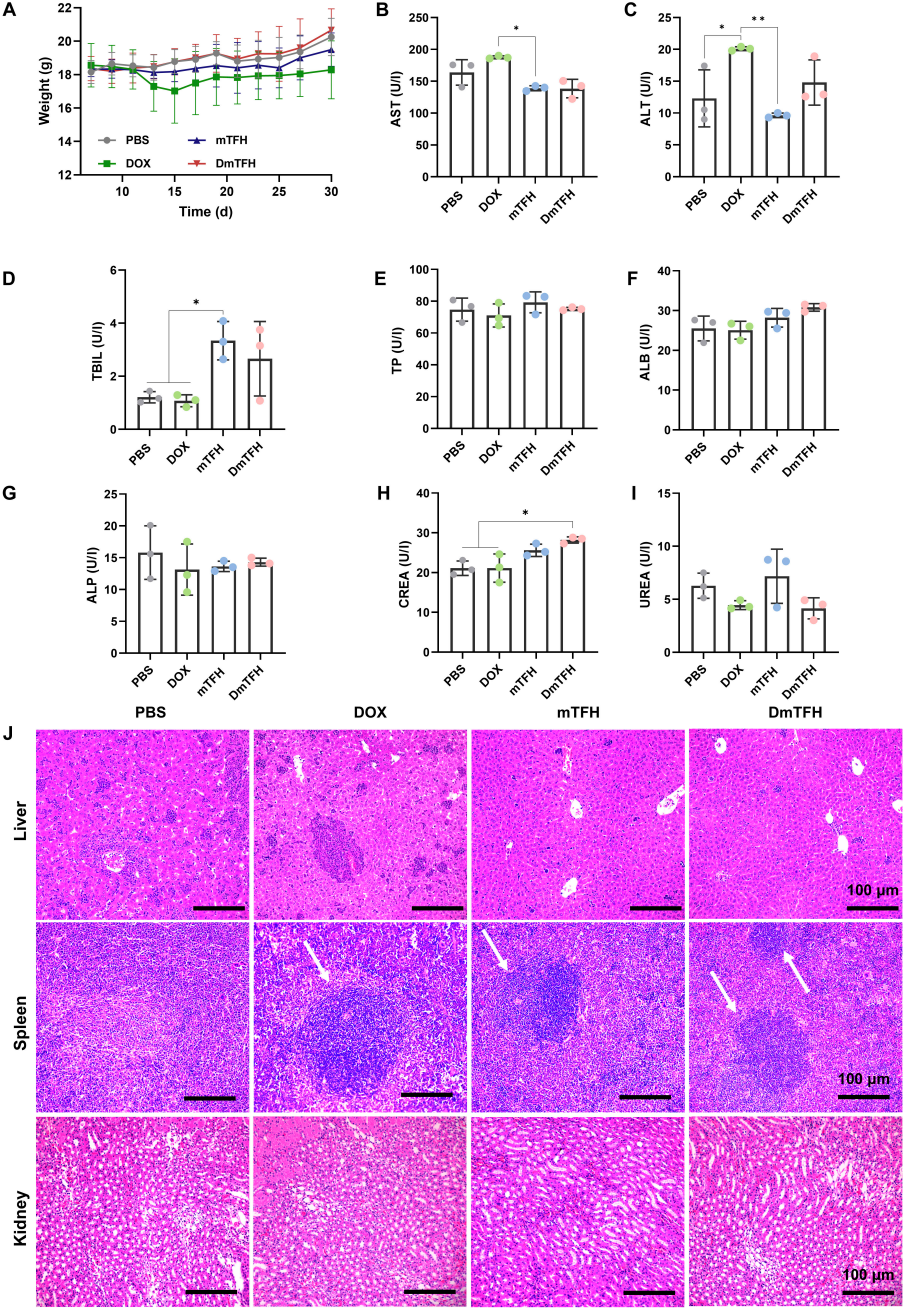
Biological safety evaluation of DmTFH. (A) Changes in body weight over time in mice administered PBS, DOX, mTFH, and DmTFH. (B to I) Serum levels of aspartate aminotransferase (AST), alanine aminotransferase (ALT), TBIL, total protein (TP), albumin (ALB), alkaline phosphatase (ALP), creatinine (CREA), and urea (UREA) in mice treated with PBS, DOX, mTFH, and DmTFH (*n* = 3). (J) H&E-stained sections of the liver, spleen, and kidney from mice treated with PBS, DOX, mTFH, and DmTFH. White arrows show the local cell density changes. Scale bars, 100 μm.

Finally, we comprehensively evaluated the biological safety of DmTFH in vivo. Throughout the treatment period, no notable weight reduction was detected in the mice from the mTFH- and DmTFH-treated groups, indicating their high biosafety. In contrast, the mice in the DOX group experienced a reduction in weight over the course of the administration, suggesting that DOX may possess some toxicity that affects the body weight of the mice (Fig. 8A). Subsequently, we observed that most of the test indexes in the blood biochemical analysis of DmTFH-treated mice showed no significant difference when compared to the control group, indicating its good blood compatibility (Fig. 8B to I). It is worth noting that treatment with mTFH and DmTFH led to slight elevations in total bilirubin (TBIL) and creatinine (CREA), implying that mTFH and DmTFH treatments may cause mild alterations in liver and kidney function due to normal metabolism in the body. Therefore, we further performed H&E staining on histological sections of primary organs. The staining results showed no significant inflammatory cell infiltration or hepatocyte necrosis in the H&E-stained liver sections of the mTFH and DmTFH groups (Fig. 8J). However, severe inflammatory cell infiltration and disordered liver tissue structure were observed in the PBS and DOX groups, indicating lesions caused by tumor development. In addition, we found varying degrees of local cell density changes indicative of immune activation in the spleen tissues of the DOX, mTFH, and DmTFH groups (as indicated by white arrows), which were consistent with the results obtained from flow cytometry. Moreover, no evident abnormal changes were observed in the spleen, kidney, and heart tissues after treatment with mTFH and DmTFH, indicating that DmTFH was a safe carrier that could mitigate DOX-induced toxicity to some extent (Fig. 8J and Fig. S14). Meanwhile, compared to free DOX, DmTFH demonstrated an enhanced T cell-mediated immune response, attributed to its intrinsic ability to activate M1 macrophages and DCs (Figs. 7A and 8J). However, the mild systemic immunosuppression caused by DOX persisted (Figs. 5E and 7A). Future research could focus on developing more precise targeted delivery systems to reduce this systemic immunosuppression, such as modifying microcapsules with targeting ligands like folic acid or RGD (arginine–glycine–aspartic acid sequence) peptides to enhance their selectivity for cancer cells.

## Conclusion

In conclusion, this study successfully developed a metal-phenolic capsule capable of synergistic drug delivery and enhanced immunomodulation by the one-step assembly of a stable MPN formed from Fe (III), TA, and HADA onto DOX-loaded Man-BSA MBs. This capsule (DmTFH) was stabilized through the coordination interactions between TA and Fe (III), as well as the conjugation of deprotonated phenolic hydroxyl groups in TA and HADA with Fe (III), leading to significantly increased drug loading capacity and preventing drug leakage under physiological conditions. The synergistic effects of DOX-induced proliferation inhibition and carrier-mediated ROS elevation enabled DmTFH, even at low drug concentrations, to exhibit significant antitumor efficacy in vitro. Moreover, by integrating the ICD effects induced by DOX and immunomodulatory capabilities mediated by mBSA, DmTFH promoted DC maturation both directly and indirectly through multiple pathways, including enhancing ICD activation in tumor cells, direct stimulation of DCs, and modulation of macrophage polarization toward the M1 phenotype. These mechanisms contributed to the enhancement of tumor-specific immune responses. In vivo evaluations using bilateral tumor models demonstrated that administration of DmTFH significantly inhibited primary and distant tumor development and markedly delayed metastasis to the lungs. Flow cytometry analysis revealed that DmTFH could induce robust local and systemic immune responses, characterized by enhanced DC maturation within lymph nodes and elevated levels of CD8^+^ and CD4^+^ T cells in both tumors and spleens. Therefore, this study presented a unique approach for developing carriers with high safety and efficiency for combined chemotherapy and immunotherapy, aiming to improve the adverse effects of chemotherapeutic agents and enhance the immunotherapeutic response through rational material design.

## Materials and Methods

### Main materials and instruments

The main materials and instruments used in the study are detailed in the Supplementary Materials.

### Preparation of mBSA MBs or DOX-loaded mBSA MBs

The mBSA solution was prepared using our previously reported wet-state heating method [9]. To load DOX in situ, varying concentrations of DOX (0.5 to 4 mg/ml) were introduced into an aqueous mBSA solution (100 mg/ml, 1 ml), and the combined solution was sonicated until the DOX was completely dissolved. Following this, the mBSA or DOX-loaded mBSA solution was subjected to heat at 75 °C for 5 min. At this point, the solution exhibited slight gelation. The solutions were then ultrasonicated for 2 min using a cell disruptor (Nanjing ATPIO Instruments Manufacture Co. Ltd.) with amplitude power set at 80%, an ultrasonic time of 3 s, and intervals of 3 s. Following centrifugation at 300*g* for 10 min, the top white foam layer was extracted, collected, and resuspended in 1 ml of MilliQ water. The centrifugation and washing processes were conducted twice (300*g*, 10 min) to eliminate any unbound DOX, and the MB dispersion was gathered for subsequent capsule preparation.

### Capsule preparation

The preparation of the capsules followed a modified procedure, as detailed in a previous study [36]. Briefly, 25 μl of TA (2 mg/ml), 50 μl of HADA (17.7 mg/ml), and 25 μl of FeCl_3_∙6H_2_O (5.7 mg/ml) solutions were sequentially added to the prepared mBSA or DOX-loaded mBSA MB solution (390 μl). To adjust the pH, 10 μl of tris (hydroxymethyl) aminomethane solution (200 mg/ml) was added to the resulting solution. After each addition of TA, HADA, FeCl_3_∙6H_2_O, and tris solutions, the suspensions were vigorously mixed for 60 s using a vortex mixer. The mixture underwent 3 washes with MilliQ water to eliminate surplus TA, HADA, FeCl_3_, and MBs. Washing steps involved centrifugation at 6,000*g* for 10 min, after which the pellet was collected. Capsules templated with mBSA MBs were named mTFH, whereas those templated with DOX-loaded mBSA MBs were named DmTFH. HADA was prepared using the previously described method, which involved coupling the carboxyl groups of hyaluronic acid with the amine groups of dopamine [9]. The DOX concentration in the final product was determined by measuring the absorbance at 484 nm and referencing it against a standard curve. Protein concentrations were assessed using a bicinchoninic acid protein assay kit, following the manufacturer’s instructions.

### In vitro drug release

The DOX-loaded microcapsule solution was suspended in PBS buffer (pH 7.4 or 5.0) and shaken at 1,000 rpm in a thermostatic shaker at 37 °C. At specific time intervals, the capsules in the buffer were centrifuged, and the supernatant (1 ml) was collected for absorbance measurement using a UV–Vis spectrophotometer. The DOX concentration in the supernatant was determined using the established standard curve. Meanwhile, 1 ml of fresh buffer was introduced into the capsule suspension to maintain a total volume of 2 ml for subsequent release studies.

### Cell experiments

#### Cell cultivation

Mouse breast cancer cells (4T1) were grown in RPMI 1640 medium with 10% fetal calf serum (BC-SE-FBS01, BioChannel Biological Technology Co. Ltd.) under normal cell culture conditions. BMDCs were isolated from the femurs and tibias of 8-week-old C57 mice, as previously reported [37].

#### Cell viability

4T1 cells were treated with varying concentrations of DOX (0.1 to 10 μg/ml), mTFH, or DmTFH (100 μl) for periods ranging from 24 to 72 h at 37 °C. When encapsulated DOX concentrations were 0.1, 1, 2.5, 5, and 10 μg/ml, the corresponding mTFH concentrations were 2.7, 27, 67, 133, and 265 μg/ml, respectively. The CCK-8 assay was used to assess cell viability. The half-maximal inhibitory concentration (IC_50_) values were determined by Prism 9 software.

#### Live/dead staining

4T1 cells were cocultured with DOX (2.5 or 10 μg/ml), mTFH (67 or 265 μg/ml), and DmTFH (67 or 265 μg/ml containing 2.5 or 10 μg/ml DOX) for either 24 or 72 h. Live or dead cells were evaluated using the Calcein-AM/PI double-staining kit, followed by visualization under a fluorescence microscope. Quantitative analysis was performed using ImageJ software.

#### Intracellular ROS detections

The DCFH-DA probe was employed to detect the intracellular ROS levels. 4T1 cells were plated in 96-well plates at a density of 2,000 cells per well and cultured for 24 h. Then, the medium containing DOX (2.5 μg/ml), mTFH (67 μg/ml), DmTFH (67 μg/ml with 2.5 μg/ml DOX), or TFH (capsules prepared using BSA MBs as templates, 67 μg/ml) was added for another 24-h incubation; untreated cells served as controls. Cells were subsequently co-incubated with PBS containing DCFH-DA in the dark for 20 min, followed by Hoechst staining. After washing to remove excess probes, fluorescence was observed under a microscope and quantified using ImageJ software.

#### Detection of markers for ICD

CRT on 4T1 cell membranes was detected by immunofluorescence staining. Cells were seeded in 96-well plates (2,000 cells/well) and cultured for 24 h, followed by treatment with DOX (2.5 μg/ml), mTFH (67 μg/ml), or DmTFH (67 μg/ml containing 2.5 μg/ml DOX) for another 24 h; untreated cells served as controls and additional negative controls (NTC) without CRT antibody incubation were included. After PBS washing, cells were fixed with 4% paraformaldehyde and blocked before overnight incubation at 4 °C with a CRT mouse monoclonal antibody (1:200 dilution, Zen-bioscience). Post-PBS washes, cells were incubated with fluorescein isothiocyanate (FITC)-conjugated goat anti-mouse immunoglobulin G (IgG) at a dilution of 1:500 for 1 h and stained with 4′,6-diamidino-2-phenylindole (DAPI) for nuclear visualization. Fluorescence microscopy was used for observation, and ImageJ for quantifying CRT fluorescence intensity. HMGB1 release was quantified using a specific ELISA kit, and the ATP release from cells was assessed by detecting the levels of extracellular ATP in the supernatant with a corresponding detection kit.

#### In vitro analysis of DC maturation and M1 macrophage polarization

To investigate the maturation of DCs induced by ICD, 4T1 cells were seeded in 6-well plates (50,000 cells/well) and cultured for 24 h, followed by treatment with DOX (2.5 μg/ml), mTFH (67 μg/ml), or DmTFH (67 μg/ml containing 2.5 μg/ml DOX) for another 24 h. The supernatants were collected, centrifuged, filtered, and supplemented with 5% fetal bovine serum to prepare conditioned medium (CM). BMDCs were cultured with this CM for 24 h before being stained with various antibodies (Fixable Viability Dye eFluor 506, anti-mouse CD11c-BV421, anti-mouse MHC II–phycoerythrin (PE)–Cy7, and CD86-AF647) and analyzed by flow cytometry. To study the direct stimulation of DmTFH on BMDCs, immature BMDCs were cultured in medium containing DOX (1 μg/ml), mTFH (27 μg/ml), or DmTFH (27 μg/ml containing 1 μg/ml DOX). After 1 day, the maturation status of BMDCs was assessed using the aforementioned antibodies and flow cytometry. For macrophage polarization experiments, RAW 264.7 cells were pretreated with IL-4 (40 ng/ml) and IL-13 (20 ng/ml) to induce an M2 phenotype. Different treatments [DOX (2.5 μg/ml), mTFH (67 μg/ml), or DmTFH (67 μg/ml containing 2.5 μg/ml DOX)] were applied to these macrophages, which were subsequently stained with Fixable Viability Dye eFluor 506, anti-mouse F4/80-BV42, anti-mouse CD11b-Super Bright 600, anti-mouse CD206-PE, and anti-mouse CD86-FITC antibodies. Flow cytometry was used to evaluate the polarization status of the macrophages. To explore the impact of macrophage polarization on BMDC maturation, CM from the differently treated macrophages were collected according to the standards for 4T1 cell CM. Subsequently, these CM were incubated together with immature BMDCs over a 24-h period, followed by flow cytometric analysis similar to the aforementioned procedure.

### Animal experiments

#### Bilateral tumor model construction

Female BALB/c mice (6 weeks old, average weight 18 ± 2 g, Vital River Laboratory Animal Technology Co. Ltd.) were used in this study, with procedures approved by the Animal Experimental Ethical Inspection Committee of Southeast University (approval no. 20250311013). A bilateral tumor model was established by injecting 2 × 10^6^ 4T1 cells in 100 μl of PBS into the right back for the primary tumor, and 5 × 10^5^ 4T1 cells in 100 μl of PBS into the left back for a distant tumor. Once the primary tumor reached about 70 mm^3^, the mice were ready for further experiments.

#### In vivo antitumor evaluation

Mice with 4T1 tumors were divided into 4 groups (*n* = 5): PBS, DOX, mTFH, and DmTFH. They received intratumoral injections of 100 μl of PBS, DOX (5 mg/ml), mTFH (15 mg/ml), or DmTFH (15 mg/ml containing 5 mg/ml DOX) every 3 d for a total of 3 treatments. Body weight and tumor size were monitored over 30 d. Tumor volume was calculated using the formula *V* = *W*^2^ × *L*/2.

#### Tissue staining analysis

After treatment, 4T1 tumor-bearing mice were euthanized, and tissues (heart, liver, spleen, lung, kidney, and tumor) were harvested. These tissues were then fixed in 4% formaldehyde and prepared for paraffin embedding, sectioning, as well as H&E staining. Histological analysis was performed using OM. For immunofluorescence, tumor sections were incubated overnight at 4 °C with anti-CRT, anti-CD4, or anti-CD8 antibodies (1:200), followed by secondary antibodies (rhodamine isothiocyanate or FITC, 1:300) for 3 h at room temperature. After washing, sections were stained with DAPI (10 μg/ml) and washed again. Tumor cell apoptosis was analyzed using a terminal deoxynucleotidyl transferase-mediated deoxyuridine triphosphate nick end labeling (TUNEL) assay kit. All fluorescent sections were scanned and analyzed.

#### Flow cytometry analysis of tumor tissues, spleens, and lymph nodes

Tumor tissues (primary tumors), spleens, and lymph nodes were collected from 4T1 tumor-bearing BALB/c mice after various treatments. The tumor tissues were cut up mechanically and then digested in a medium containing 1 mg/ml collagenase IV, 0.2 mg/ml deoxyribonuclease I, and 0.2 mg/ml hyaluronidase at 37 °C for 1 to 1.5 h to obtain single-cell suspensions of tumor tissues. Single-cell suspensions from spleens and lymph nodes were obtained by grinding these tissues, followed by filtration through cell strainers (Beckman, 40 μm). The blood lysis buffer was used for red blood cell lysis for 10 min at room temperature. After lysis, the cell suspensions were treated with FcR blocking buffer at room temperature for 10 min. For the analysis of mature DCs in lymph nodes, single-cell suspensions were incubated with Fixable Viability Dye eFluor 506, anti-mouse CD45–allophycocyanin (APC)–Cy7, anti-mouse CD11c-BV42, anti-mouse MHC II-PE-Cy7, and CD86-AF647 antibodies. To detect helper T cells (CD3^+^CD4^+^) and cytotoxic T cells (CD3^+^CD8^+^) in the spleen, single-cell suspensions were incubated with Fixable Viability Dye eFluor 506, anti-mouse CD45-APC-Cy7, CD3e-FITC, CD4-BV786, and CD8-Percp-Cy5.5 antibodies. For the detection of M1-polarized macrophages and activated T cells in tumors, single-cell suspensions were incubated with Fixable Viability Dye eFluor 506, anti-mouse CD45-APC-Cy7, CD3e-FITC, CD4-BV786, CD8-Percp-Cy5.5, CD11b-Super Bright 600, F4/80-BV421, CD206-PE, and CD86-AF647 antibodies.

#### ELISA analysis

The levels of IL-6, IFN-γ, and TNF-α in serum were assessed via ELISA in accordance with the manufacturer’s instructions provided with the ELISA kit.

#### Analysis of biochemical markers in the blood

To evaluate the biosafety of the carriers, we collected blood samples from mice after various treatments and performed tests for biochemical markers.

#### Statistical analysis

Data are presented as mean ± SD. Statistical analyses were conducted using GraphPad Prism 9. Data normality was assessed with the Shapiro–Wilk test, and variance homogeneity with the *F* test or Bartlett’s test. Differences between the 2 groups were analyzed using a 2-tailed *t* test for normal distributions or the Mann–Whitney test for non-normal distributions. For multiple comparisons, one-way analysis of variance (ANOVA) was used for normal data and the Kruskal–Wallis test for non-normal data. All analyses were performed with at least 3 replicates (*n* ≥ 3) to ensure reliability. Significance levels: ns (*P* > 0.05), * (*P* < 0.05), ** (*P* < 0.01), *** (*P* < 0.001), **** (*P* < 0.0001).

## Data Availability

The data are available from the corresponding authors upon a reasonable request.
