# Supplementary material for: Microbubble-Templated Immunoactive Metal-Phenolic Capsules for Drug Delivery and Enhanced Cancer Immunotherapy
Source: Research (Wash D C). 2025 Jul 4;8:0752. doi: 10.34133/research.0752 (PMC12231236; doi:10.34133/research.0752)
Supplement: Supplementary 1 — Supplementary Text Figs. S1 to S14 Table S1 [file research.0752.f1.docx]

**Supporting Information**

**Microbubble-Templated Immunoactive Metal-Phenolic Capsules for Drug Delivery and Enhanced Cancer Immunotherapy**

Xin Tan ^1^, Xiaojing Wu ^2^, Renwang Sheng ^3^, Yinghua Tao ^1^, Weikun Li ^1^, Yanling Liang ^1^, Bo Gui ^4^, Huiqin Lu ^2^, Diyi Feng ^1^, Nuoya Chen ^1^, Fangzhou Liu *^4^, Ling Liu *^2^, Liqin Ge *^1,5^

^1^ State Key Laboratory of Digital Medical Engineering, School of Biological Science and Medical Engineering, Southeast University, Nanjing 210096, P.R. China.

^2^ Jiangsu Provincial Key Laboratory of Critical Care Medicine, Department of Critical Care Medicine, Zhongda Hospital, School of Medicine, Southeast University, Nanjing 210009, P.R. China.

^3^ School of Medicine, Southeast University, Nanjing 210009, P.R. China.

^4^ Department of Head & Neck Surgery, Jiangsu Cancer Hospital & Jiangsu Institute of Cancer Research & The Affiliated Cancer Hospital of Nanjing Medical University, Nanjing 210029, P.R. China

^5^ Advanced Ocean Institute of Southeast University, Nantong 226000, P.R. China.

^*^ Corresponding Author.

Email: lqge@seu.edu.cn (Liqin Ge); liulingdoctor@126.com (Ling Liu); liufangzhou@njmu.edu.cn (Fangzhou Liu)

**Main materials**

Doxorubicin hydrochloride (DOX.HCl, D107159), tannic acid (TA, T103998), D-(+)-mannose (M103969), dopamine hydrochloride (D103111), N-Hydroxysuccinimide (NHS, H109330), N-(3-Dimethylaminopropyl)-N’-ethylcarbodiimmide hydrochloride (EDC, E106172), iron (III) chloride hexahydrate (FeCl_3_·6H_2_O, F419646), potassium bromide (P116273) and Tris(hydroxymethyl)aminomethane (T591027) were purchased from Aladdin Biochemical Technology Co. Ltd., China. The bovine serum albumin (BSA, V900933) and collagenase IV (C5138) were bought from Sigma-Aldrich Chemicals Reagent Co. Ltd., USA. Hyaluronic acid (HA, MW = 400000, RH445435) was obtained from Rhawn Chemicals Reagent Co. Ltd., China. HCl (10011018) was procured from Sinopharm Chemical Reagent Co. Ltd., China. Calcein-AM/PI double staining kit (KGA9501), reactive oxygen species assay kit (KGA7308), and CCK-8 kit (KGA9305) were all obtained from KeyGEN Biotechnology Co. Ltd., China. Triton X-100 (P0096), Hoechst 33342 (C1022), QuickBlock™ blocking buffer (P0260), FITC-conjugated goat anti-mouse IgG (H + L, A0568), 4',6-diamidino-2-phenylindole (DAPI) staining solution‌‌ (C1005), Enhanced ATP assay kit (S0027), CD4 mouse monoclonal antibody (AG1393), CD8 alpha rabbit monoclonal antibody (AG1414), and Penicillin-streptomycin-gentamicin solution (C0224) were bought from Beyotime Biotechnology Co. Ltd., China. Calreticulin (6H1) mouse mAb (220189) was purchased from Zen-bioscience Biotechnology Co., Ltd., China. HMGB1 ELISA kits, Dulbecco’s modified Eagle’s medium (DMEM), Phosphate buffered saline (PBS, pH 7.4), RPMI-1640 medium, fetal calf serum (FBS, BC-SE-FBS01), 4% paraformaldehyde and bicinchoninic acid (BCA) protein assay kit were obtained from BioChannel Biological Technology Co. Ltd., China. The IL-6, IFN-γ and TNF-α ELISA kits were purchased from Shanghai Jianglai Biotechnology Co., Ltd., China. Murine GM-CSF (315-03), IL-4 (214-14), and IL-13 (210-13) were purchased from PeproTech, USA. DNase I (1121MG010) was purchased from Biofroxx, Germany. Hyaluronidase (H792266) was obtained from Macklin Biotech Co., Ltd., China. The blood lysing reagent (555899) was acquired from Becton, Dickinson and Company (BD). The FcR blocking reagent (mouse, 130092575) came from Miltenyi Biotec, Germany. Antibodies used for flow cytometry were purchased from Thermo Fisher Scientific, BD Biosciences. The detailed information was listed in the below: Fixable Viability Dye eFluor 506 (Thermo, 65-0866-14), anti-mouse CD11c-BV421 (Clone: N418; 117329; 1:100; BioLegend), anti-mouse MHC II-PE-Cy7 (Clone: AF6-120.1; 116420; 1:100; BioLegend), and CD86-AF647 (Clone: GL-1; 105019; 1:200; BioLegend), anti-mouse F4/80-BV421 (Clone: BM8; 123132; 1:200; BioLegend), anti-mouse CD11b-Super Bright 600 (Clone: M1/70; 63-0112-82; 1:200; Invitrogen), anti-mouse CD206-PE (Clone: C068C2; 141706; 1:100; BioLegend), anti-mouse CD86-FITC (Clone: GL1; 561962; 1:200; BD Biosciences), anti-mouse CD45-APC-Cy7 (Clone: 30-F11; 557659; 1:200; BD Biosciences), CD3e-FITC (Clone: 145-2C11; 553061; 1:200; BioLegend), CD4-BV786 (Clone: H129.19; 740873; 1:200; BioLegend), and CD8-Percp-Cy5.5 (Clone: 53-6.7; 551162; 1:100; BioLegend) antibodies.

**Instruments**

The capsules' morphology was characterized using optical microscopy (OM, MF52, Guangzhou Mingmei Optoelectronics Technology Co., Ltd), scanning electron microscopy (SEM, Zeiss 11003457, Ultra Plus, Germany), transmission electron microscopy (TEM, Thermoscientific), atomic force microscopy (AFM), and confocal laser scanning microscopy (CLSM, Leica TCS SP8). The energy-dispersive X-ray spectroscopy (EDX) mapping data of the capsules were obtained from TEM. UV-Vis absorption spectra were measured using a UV spectrophotometer (HTH instrument), and the peak positions were determined by fitting the data to a Gaussian function. Zetasizer Nano ZSE (Malvern) was used for detecting the Zeta potential and size distribution of the capsules in water. ^1^H NMR spectra were recorded at room temperature on an AVANCE III HD 600 MHz spectrometer (Bruker) using deuterated water as the solvent. Fourier-transform infrared spectroscopy (FTIR) was employed to obtain the infrared spectra of the capsules. For secondary structure assessment, the amide I region (1700-1600 cm^-1^) was analyzed using the second derivative method with Origin 2024 software (OriginLab, Northampton), and peak positions in the spectra were fitted using Gaussian functions. The fluorescence microscope was used to capture cellular fluorescence images. Flow cytometry was performed using a full-spectrum profiling flow cytometer (Cytek Biosciences). All samples were prepared according to standard protocols.

**
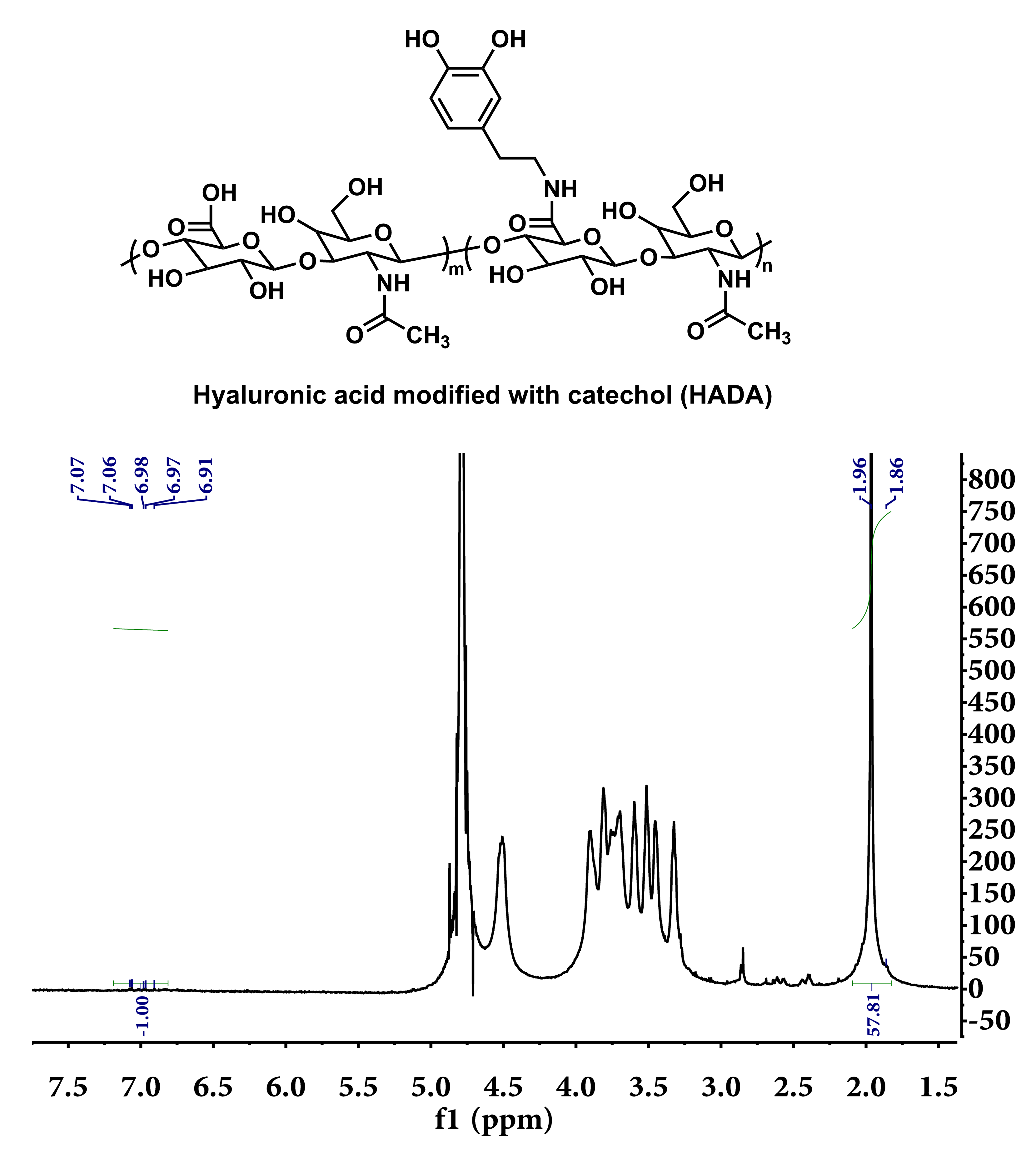
**

**Figure S1.** Schematic diagram of the chemical structure of hyaluronic acid modified with catechol (HADA) and ^1^H NMR spectrum of HADA. The grafting rate of catechol group on HA was about 1.7%.


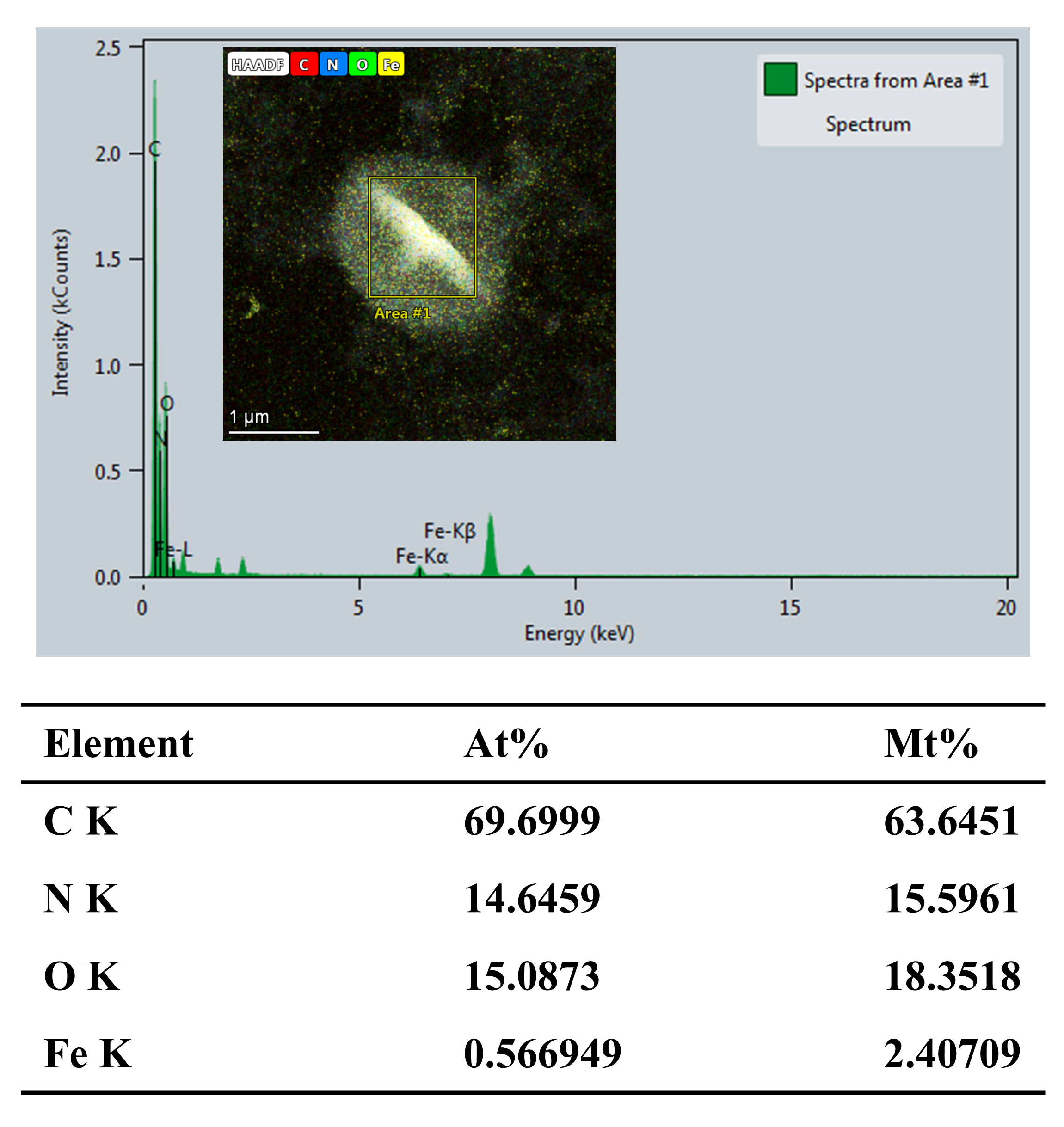


**Figure S2.** Energy-dispersive X-ray (EDX) spectroscopy mapping and corresponding elemental quantitative analysis of DmTFH.


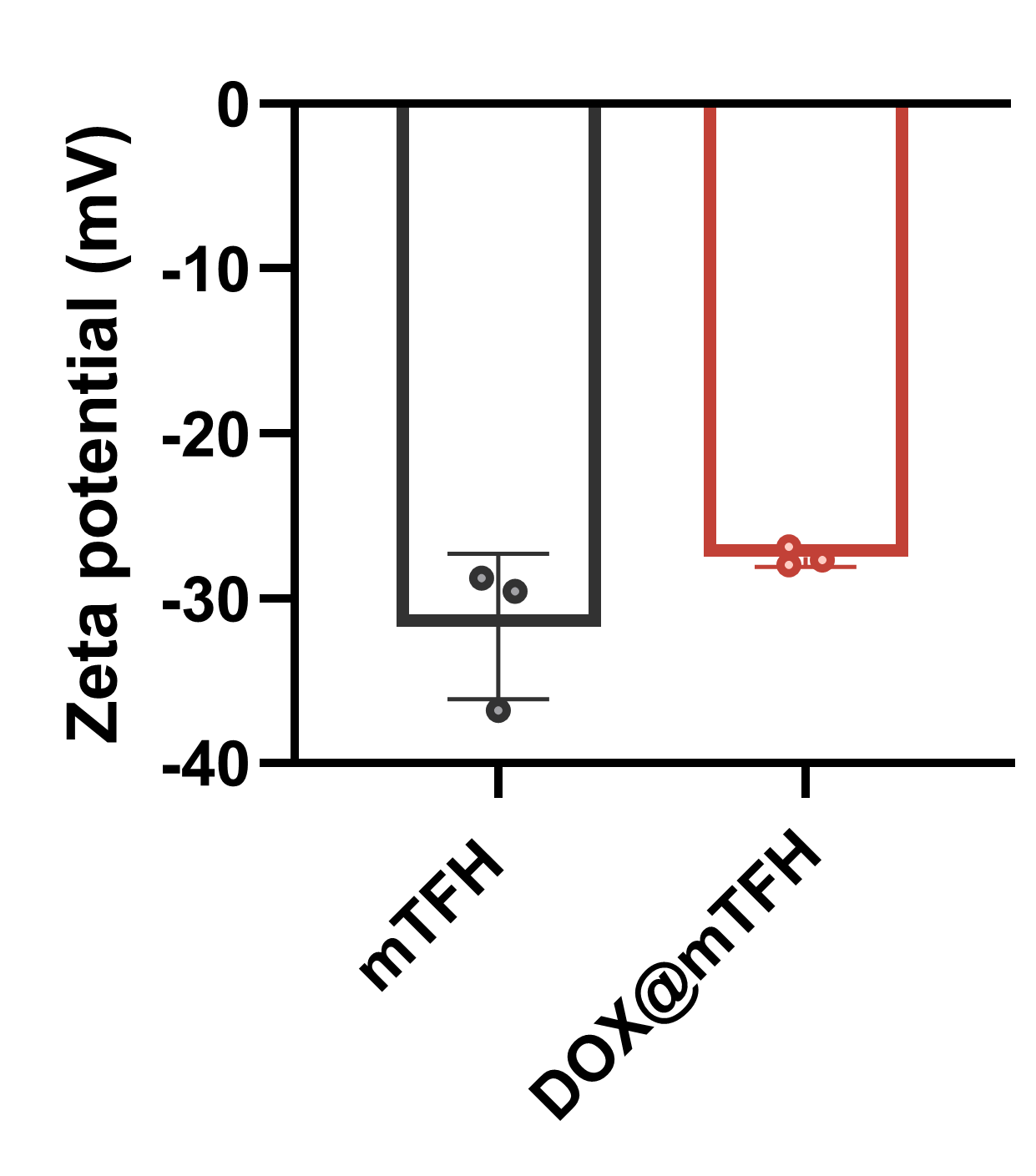


**Figure S3.** Zeta potential of mTFH and DmTFH.


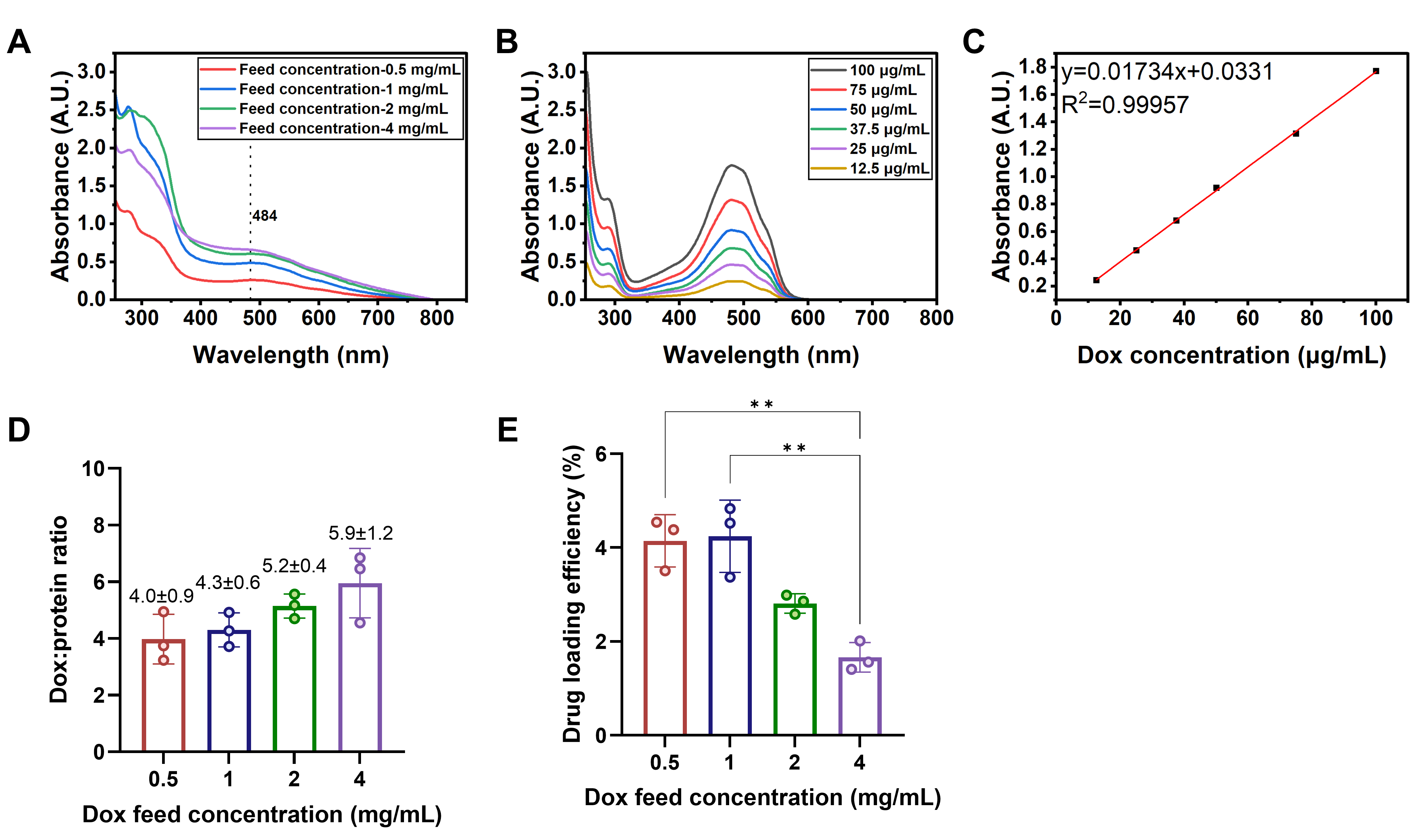


**Figure S4.** (A) UV-Vis spectrum of DmTFH obtained at different DOX feed concentrations (0.5, 1, 2, and 4 mg/mL). (B) UV-Vis spectrum of DOX at different concentrations (12.5, 25, 37.5, 50, 75, and 100 μg/mL). (C) Standard curve of DOX absorbance versus concentration based on the absorbance values at 484 nm from (B). (D) Relationship between different DOX feed concentrations and the drug loading capacity (molar ratio of DOX to BSA protein) of the resulting DmTFH. (E) Relationship between different DOX feed concentrations and the drug loading efficiency of the resulting DmTFH.


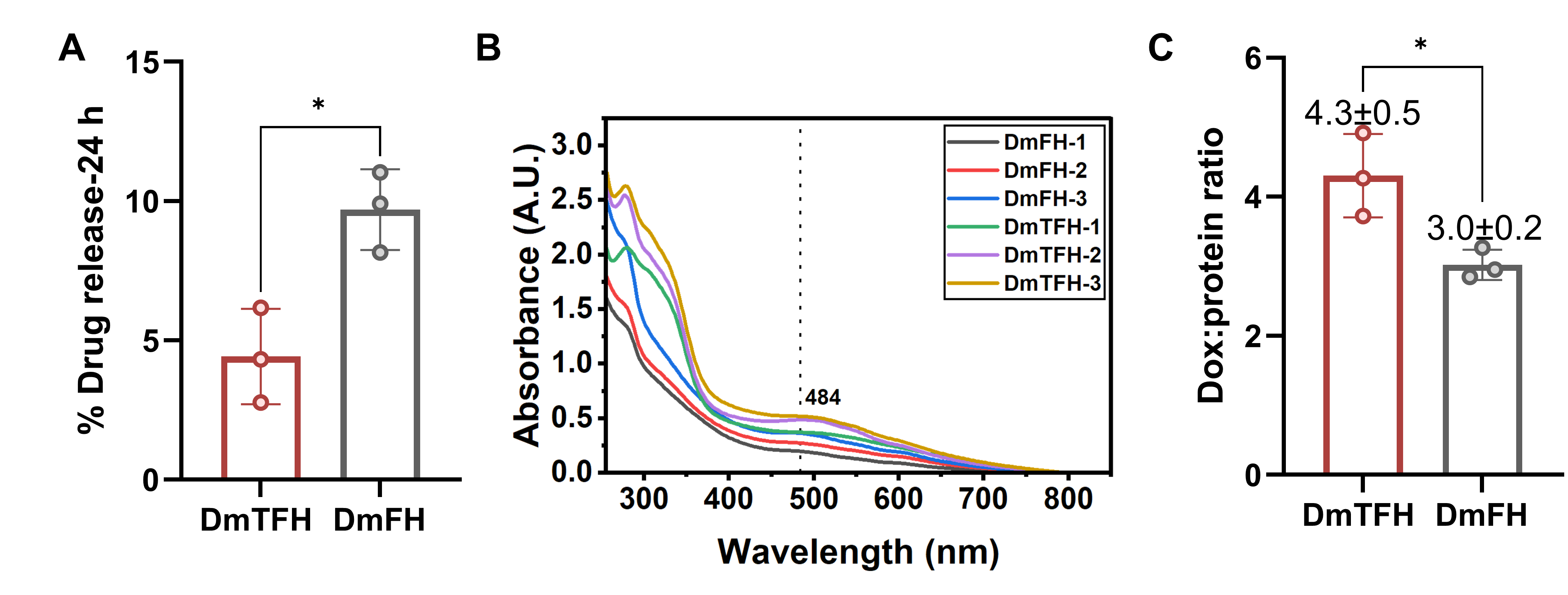


**Figure S5.** (A) The percentage of drug released after 24 h in PBS for both DmTFH and DmFH formulations. (B) UV-Vis spectra of DmTFH and DmFH obtained at DOX feed concentrations of 1 mg/mL (n = 3). (C) The comparison of the drug loading capacity (molar ratio of DOX to BSA protein) between DmTFH and DmFH formulations at DOX feed concentrations of 1 mg/mL.


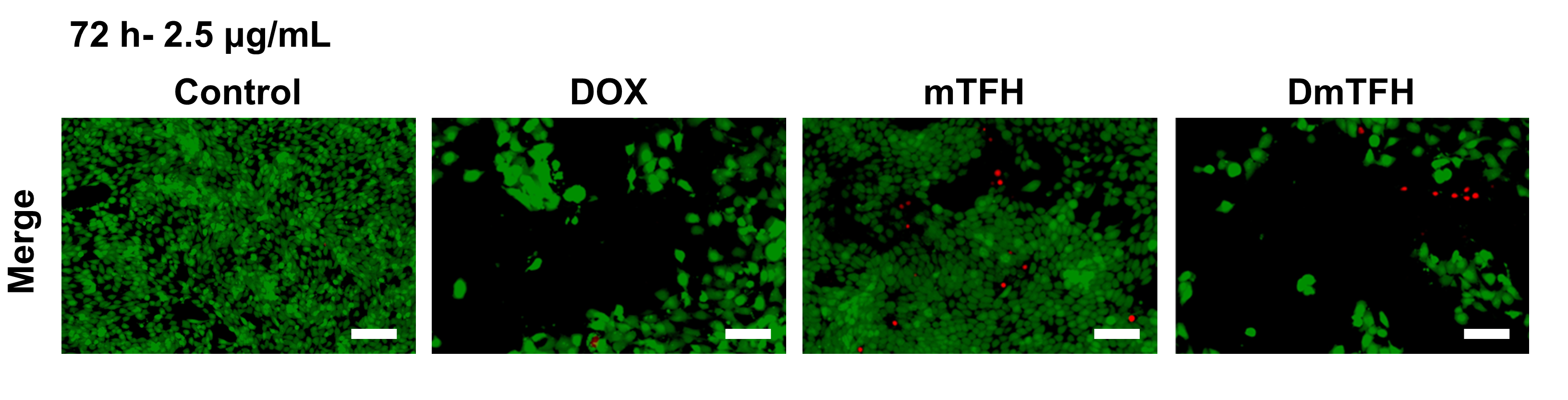


**Figure S6.** Images of live/dead staining of 4T1 cells following a 72-h incubation with free DOX, mTFH, and DmTFH at corresponding concentrations for 72 h. Control represents untreated cells. Green and red fluorescence indicate live cells and dead cells, respectively. Scale bar = 100 μm.


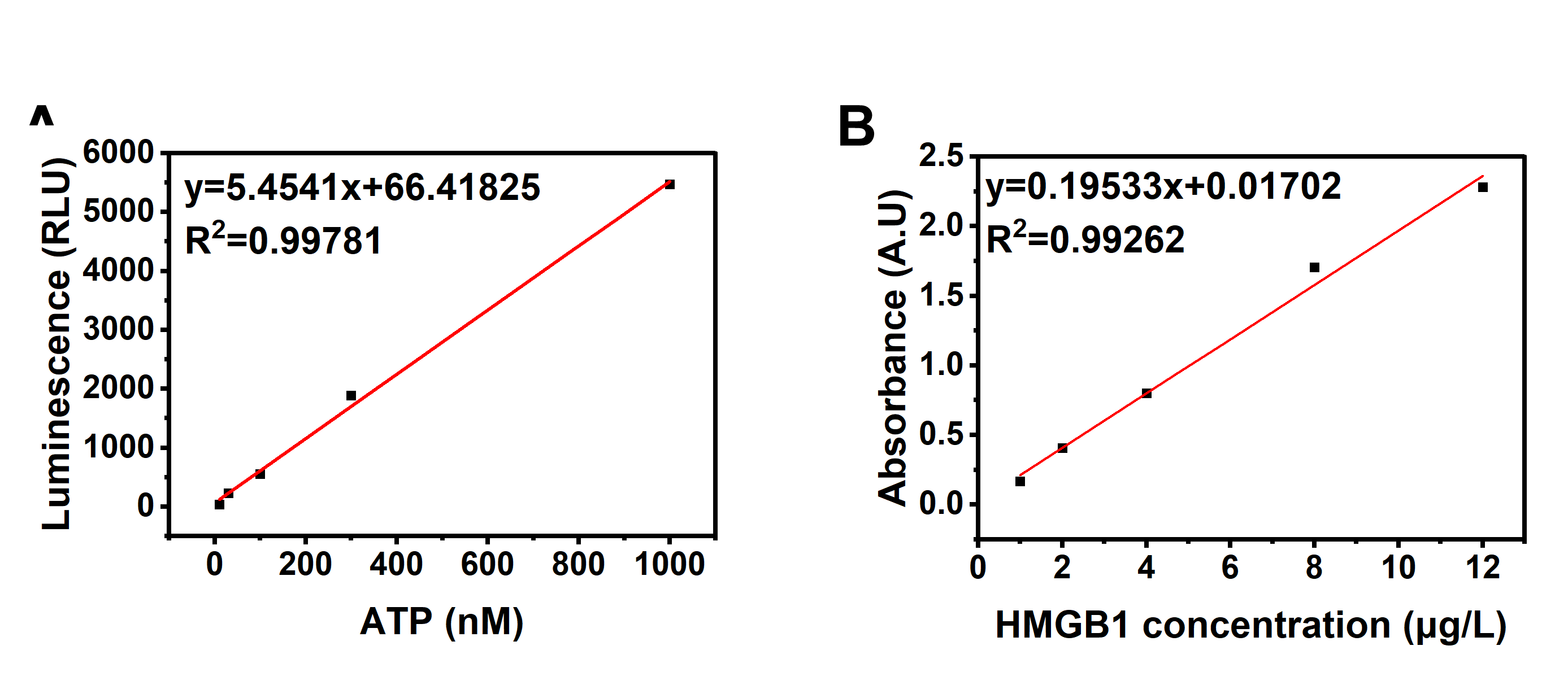


**Figure S7.** (A) The standard curve for ATP concentration determined by luminometer. (B) The standard curve for HMGB1 concentration determined by mouse HMGB1 ELISA kit.


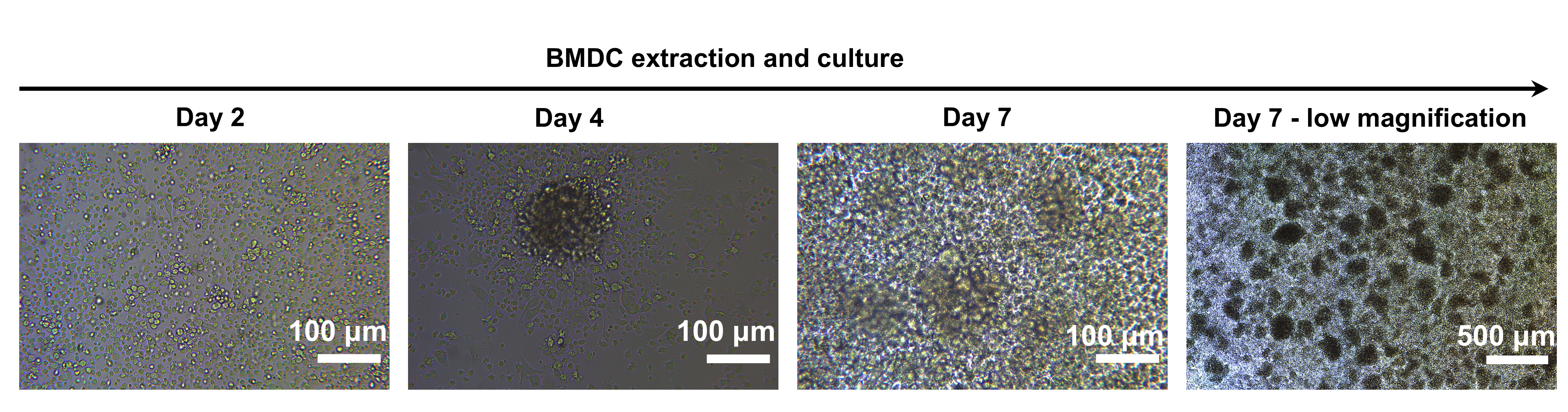


**Figure S8.** The progression of bone marrow-derived dendritic cell (BMDC) culture over time.


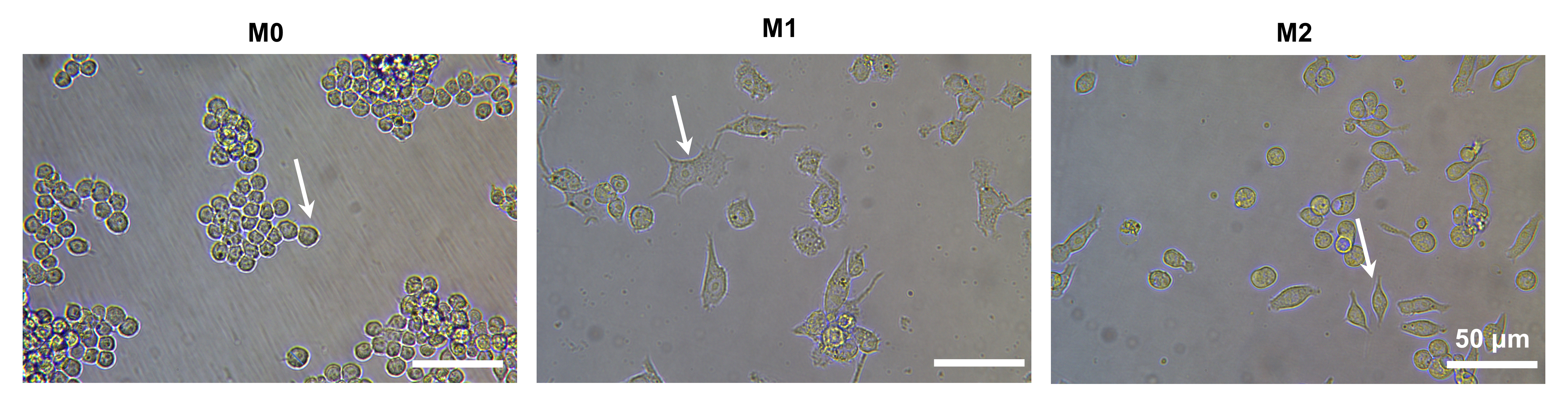


**Figure S9.** The morphological changes of RAW 264.7 cells under different polarization states (M0, M1, and M2). The arrow represents the corresponding polarization states.


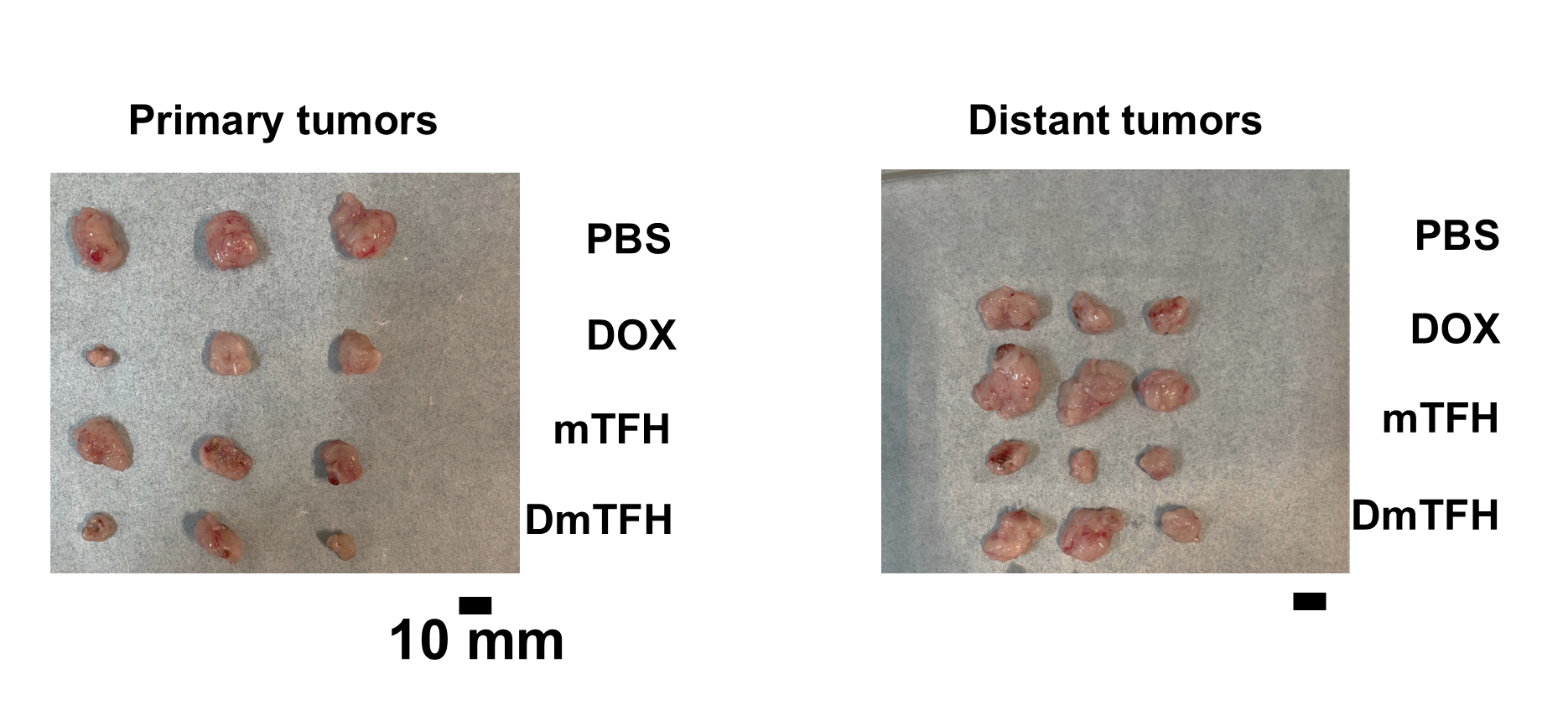


**Figure S10.** Final images of the excised primary and distant tumors.


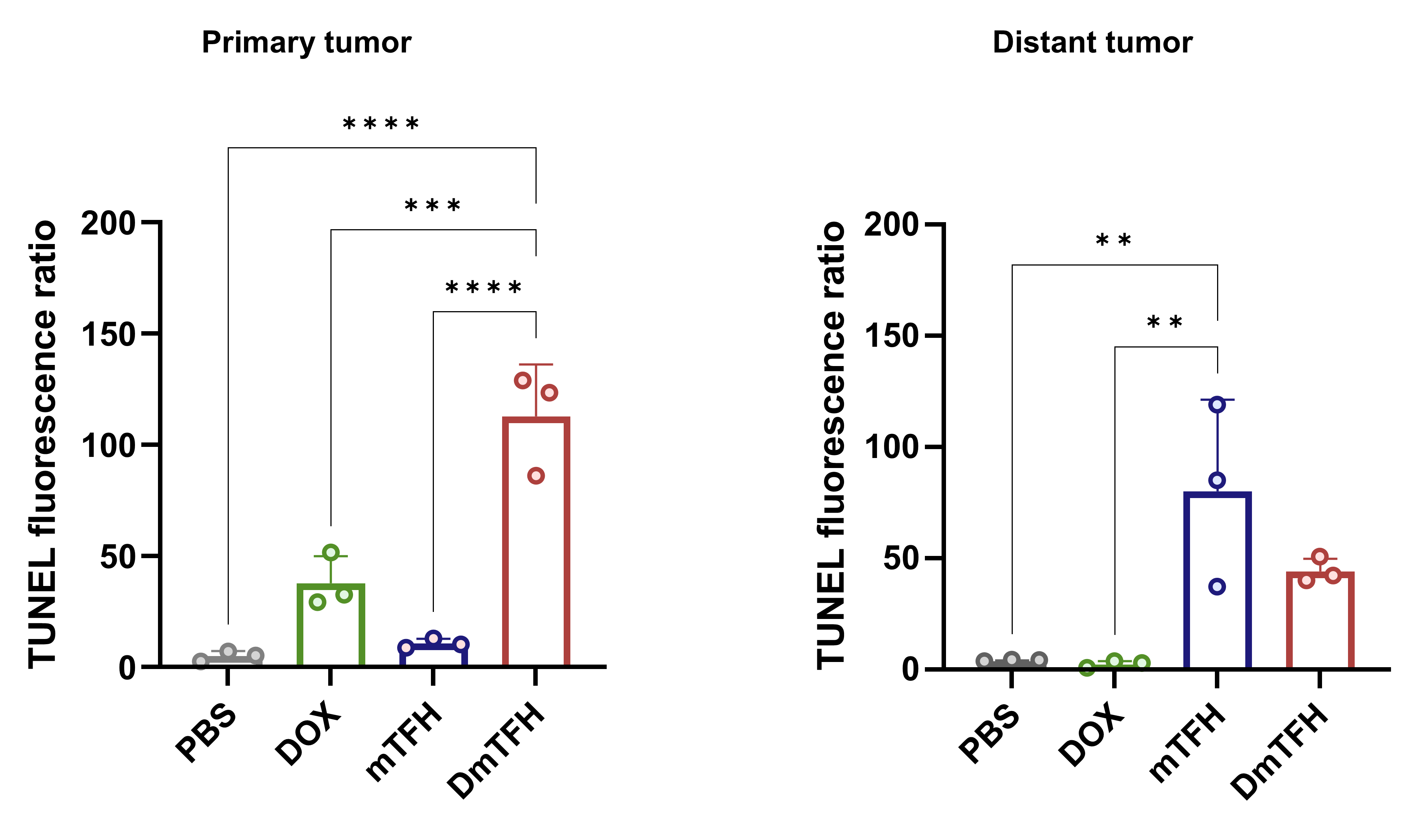


**Figure S11.** The corresponding quantification analysis of the TUNEL staining results.


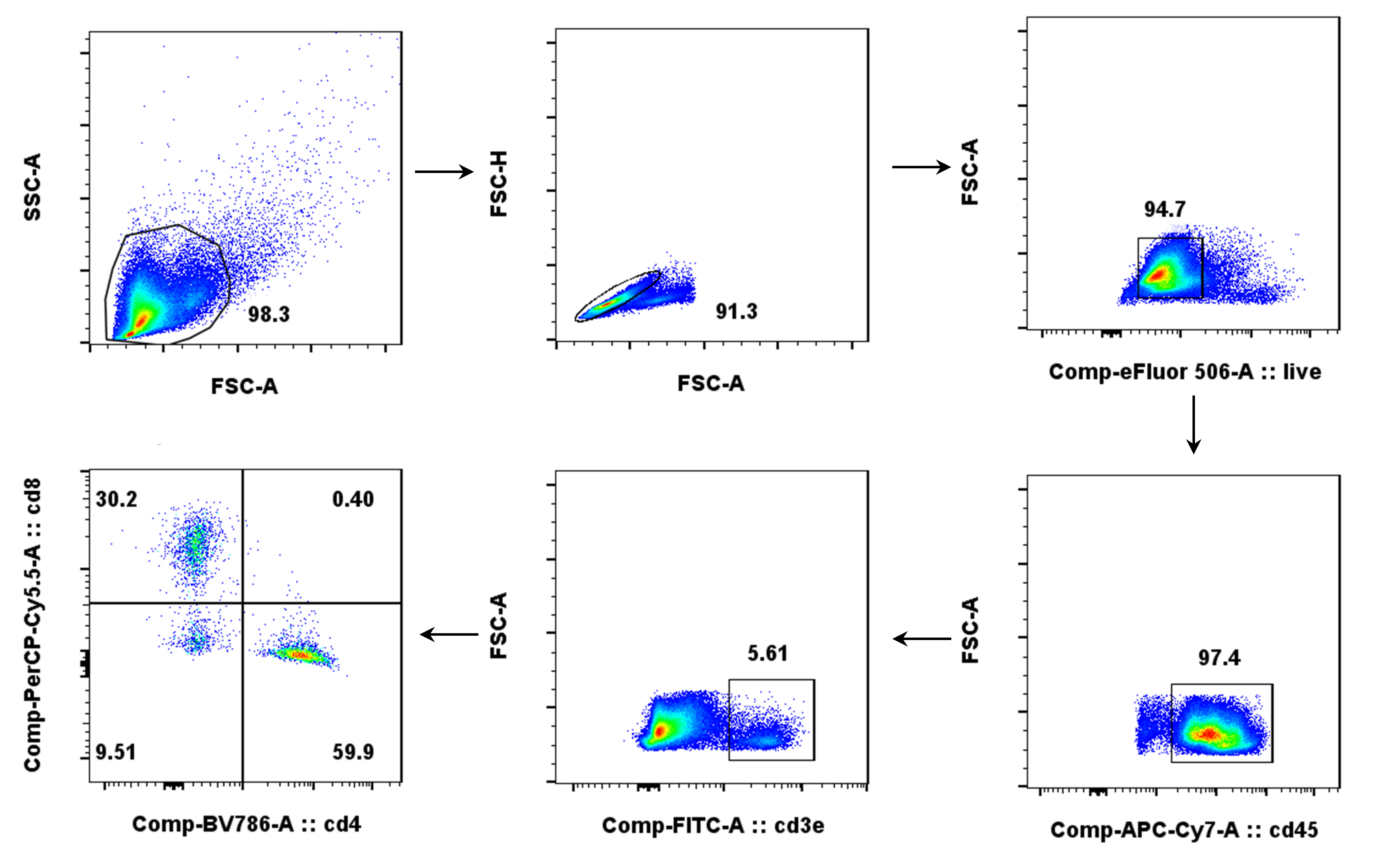


**Figure S12.** The flow cytometric gating strategy of CD4^+^ and CD8^+^ T cells in spleen.


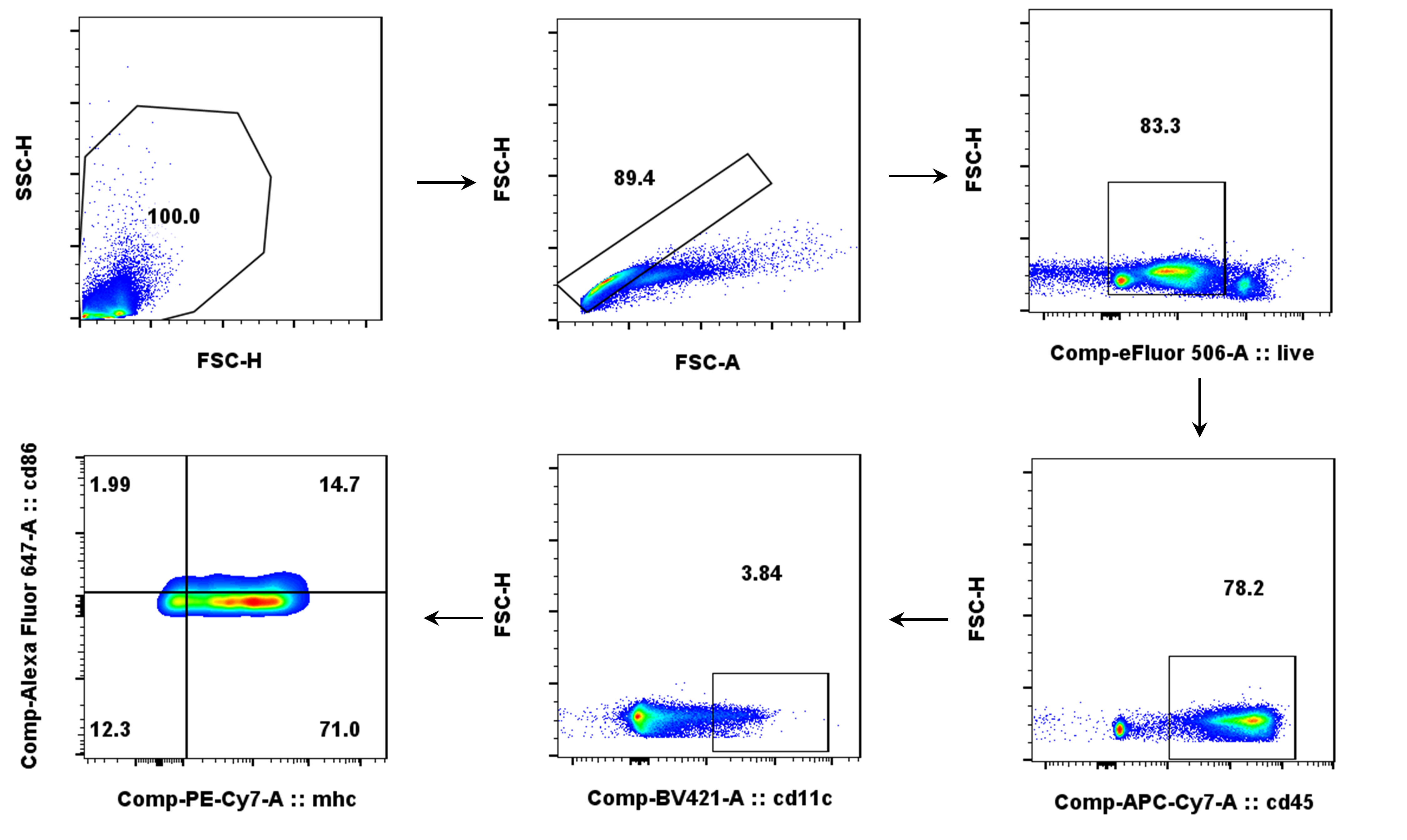


**Figure S13.** The flow cytometric gating strategy of CD86^+^ MHC II^+^ matured DC in lymph nodes.


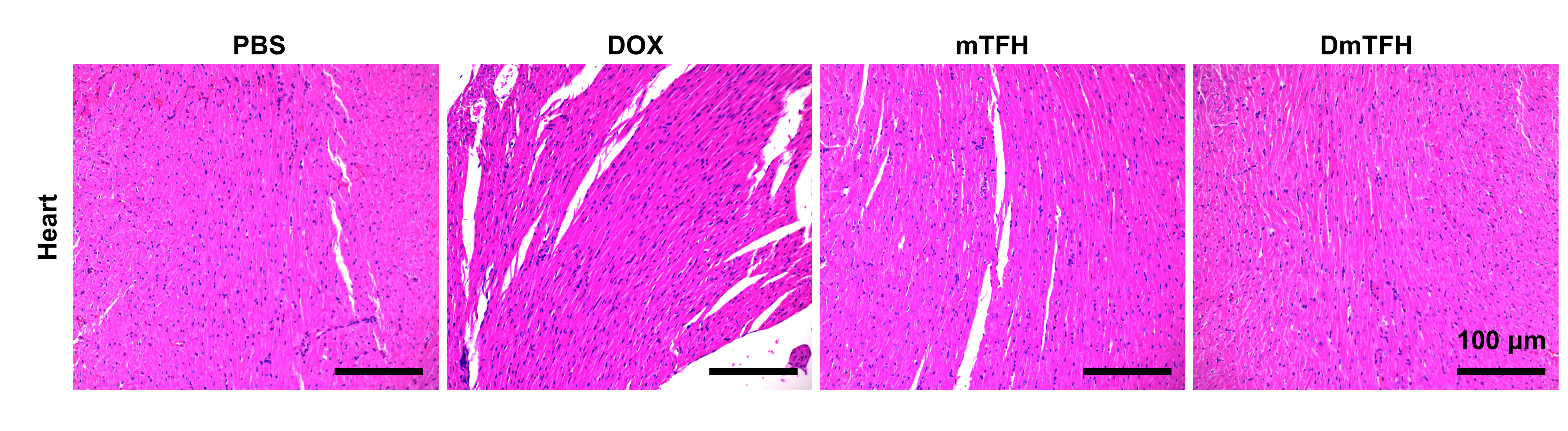


**Figure S14.** Histological sections of heart from different groups were stained with hematoxylin and eosin (H&E). The images show representative sections from each group. Scale bars represent 100 μm.

**Table S1.** The secondary structure percentages of mTFH and DOX@mTFH

| Wavenumber  (cm^-1^) | mTFH | DOX@mTFH |
| --- | --- | --- |
| (1610-1621)  Intermolecular β-sheet | 22.05417% | 29.56311% |
| (1623-1636)  β, extended | 11.73897% | 5.73397% |
| (1642-1647)  Random coil | 9.34105% | 0.19174% |
| (1650-1655)  α-helix | 37.25294% | 43.81203% |
| (1662-1674)  turn | 12.90462% | 13.7239% |
| (1680-1690)  β-turn | 5.20836% | 5.48803% |
